# Massive wildfires followed oceanic anoxic events during the Late Devonian Frasnian-Famennian mass extinction

**DOI:** 10.1126/sciadv.ady4534

**Published:** 2026-04-01

**Authors:** Man Lu, Yongge Sun, Guoqiang Duan, Takehito Ikejiri, Naihao Liu, Qingyong Luo, Dawei Lv, Richard Carroll, Yuehan Lu

**Affiliations:** ^1^ Hainan Institute of China University of Petroleum (Beijing), Sanya 572025, China.; ^2^ State Key Laboratory of Petroleum Resources and Engineering, China University of Petroleum (Beijing), Beijing 102249, China.; ^3^ College of Geoscience, China University of Petroleum (Beijing), Beijing 102249, China.; ^4^ Molecular Eco-Geochemistry (MEG) Laboratory, Department of Geological Sciences, The University of Alabama, Tuscaloosa, AL 35487, USA.; ^5^ Organic Geochemistry Unit, Key Laboratory of Geoscience Big Data and Deep Resource of Zhejiang Province, School of Earth Sciences, Zhejiang University, Hangzhou 310027, China.; ^6^ Alabama Museum of Natural History, The University of Alabama, Tuscaloosa, AL 35485, USA.; ^7^ School of Information and Communications Engineering, Xi'an Jiaotong University, Xi'an 710049, China.; ^8^ College of Earth Sciences and Engineering, Shandong University of Science and Technology, Qingdao 266590, China.; ^9^ Energy Investigation Program, Geological Survey of Alabama, Tuscaloosa, AL 35401, USA.

## Abstract

Extensive wildfire profoundly influences Earth system feedback and can drive major ecosystem disturbances, yet its timing and role in the Late Devonian Frasnian-Famennian (F-F) mass extinction remain unclear. To determine whether wildfires were a driver or consequence of contemporaneous oceanic anoxic events (OAEs), we present a high temporal resolution multiproxy record (biomarkers, microfossils, and trace metals) from the Chattanooga Shale in the southeastern United States. Pyrogenic PAHs and inertinite macerals increased after peaks in δ^13^C_org_ and redox-sensitive proxies, indicating that wildfire activity intensified in response to post-OAEs oxygen rise rather than triggering anoxia. Modeling of global δ^13^C records reveals that the lag between OAEs/organic carbon burial and wildfire onset reflects the time required for atmospheric oxygen to accumulate to levels sustaining widespread combustion. Together, these results provide the first high-resolution dataset capable of resolving the temporal sequence between OAEs and wildfire activity, enabling the establishment of their causal linkage during the catastrophic F-F environmental disruptions.

## Teaser

Wildfire was a consequence — not a cause — of widespread marine anoxia during the Late Devonian mass extinction.

## INTRODUCTION

Wildfires are an integral component of Earth system. They play an important role in the development and maintenance of varied types of landscapes and biomes that are important for the sustenance of biodiversity (*1*). On a human scale, extreme wildfires have substantial economic, social, and environmental impacts, with concerns that their frequency, severity, and extent are increasing across the globe (*2*). On the geological time scale, wildfires have the potential to act as a primary force or catalyst in altering and influencing the evolutionary trajectory of Earth’s ecosystems. For example, increased wildfire activity has been linked to contemporaneous biotic crises, such as the Permian-Triassic event (*3*), Triassic-Jurassic boundary mass extinction (*4*, *5*), Jurassic Toarcian oceanic anoxic event (T-OAE) (*6*), and Cretaceous OAE2 (*7*). Understanding the role that wildfires have played in key life events in Earth’s history is crucial for unraveling their functioning mechanisms within the Earth system, which is important for addressing the current challenges posed by global environmental changes.

Beginning in the Late Silurian, the evidence of paleo-wildfires [e.g., fossil charcoals and pyrogenic polycyclic aromatic hydrocarbons (PAHs)] was consistently present in the geological records, spanning almost entire Phanerozoic era (*8*). Current evidence points to a spatial-temporal rise in wildfire occurrences during the Late Devonian period (*9*–*13*). This wildfire surge has been hypothesized to mobilize and increase the flux of terrestrial nutrients into oceans, fueling primary production, initiating marine anoxia, and ultimately leading to the marine biotic crisis that occurred during the Late Devonian (*10*, *14*). Conversely, global wildfires can also be a consequence of marine anoxia. The Late Devonian Frasnian-Famennian (F-F) boundary is associated with widespread OAEs (namely, Kellwasser events) (*15*), which, along with temperature changes, are widely regarded as the proximate cause of severe marine biodiversity losses across this interval [(*16*) and references therein]. The OAEs have been globally documented by depositions of black shales (*17*, *18*) and positive δ^13^C excursions of organic and inorganic carbon (δ^13^C_org_ and δ^13^C_carb_) (*19*, *20*). These characteristics suggest enhanced marine organic carbon (OC) burial, driven by primary productivity and marine anoxia, could lead to a rise in the atmospheric oxygen level (*p*O_2_) (*21*), thereby increasing the likelihood of wildfire occurrence (*22*). To resolve whether wildfires served as a trigger or a consequence of the Late Devonian marine anoxia, it is essential to establish a record that not only captures wildfires and marine anoxia concurrently but also has sufficient resolution to resolve their temporal sequences throughout the Late Devonian mass extinction interval. Such a high-resolution, integrated record remains unavailable to date.

**Fig. 1.**
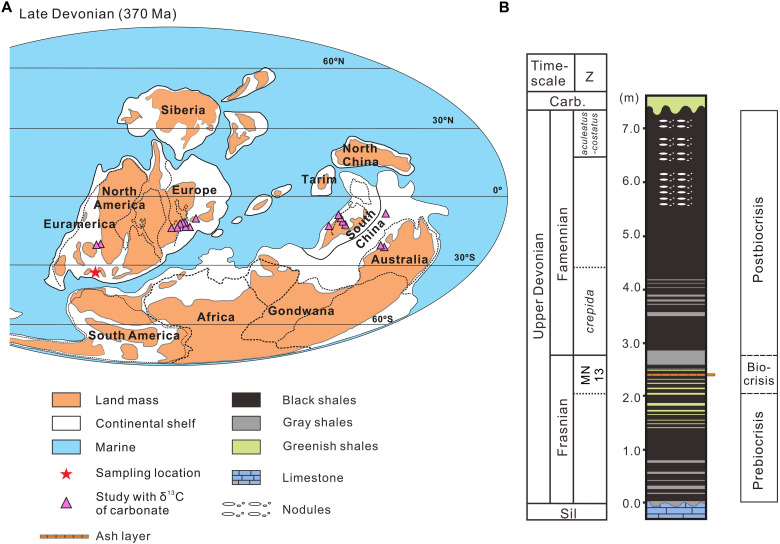
Late Devonian paleo-map of the sampling locality and stratigraphy for the Chattanooga Shale in Chestnut Mound, central Tennessee, southeastern United States. (**A**) Global paleogeographic map showing localities of the study section and global carbonate δ^13^C (δ^13^C_carb_) records synthesized from the literatures. Red star denotes the present study site, the Chestnut Mound outcrop of the Chattanooga Shale; yellow triangles denote the sections with δ^13^C_carb_ records published previously. The paleogeographic map (370 Ma) was adapted and reprinted from (*99*), copyright 2020, with permission from Elsevier. (**B**) Stratigraphic column of the Chattanooga Shale was modified and reprinted from (*77*), copyright 2021, with permission from Elsevier. Conodont zonation was reprinted from (*59*), copyright 2015, with permission from Cambridge University Press, with modifications based on (*100*). Z: conodont zonation.

In this study, we present a high temporal resolution (1-cm interval) dataset from the F-F biocrisis interval of the Chattanooga Shale, which was deposited in an epicontinental sea from the southern Appalachian Basin (Fig. 1) (*23*). The F-F mass extinction represents one of the five greatest Phanerozoic biotic crises (*24*). We collected a suite of geochemical proxies of the Chattanooga Shale exposed in the Chestnut Mound outcrop (central Tennessee, USA), including molecular biomarkers, inertinite macerals, stable carbon isotopes of OC (δ^13^C_org_), total mercury (Hg) contents, and trace metals. This multiproxy record establishes a clear temporal sequence between wildfire surges and OAEs, thereby allowing elucidating their causal relationships and shedding light on the specific role that wildfires had played during the Late Devonian mass extinction.

## RESULTS AND DISCUSSION

### Evidence for wildfires during the F-F mass extinction

**Fig. 2.**
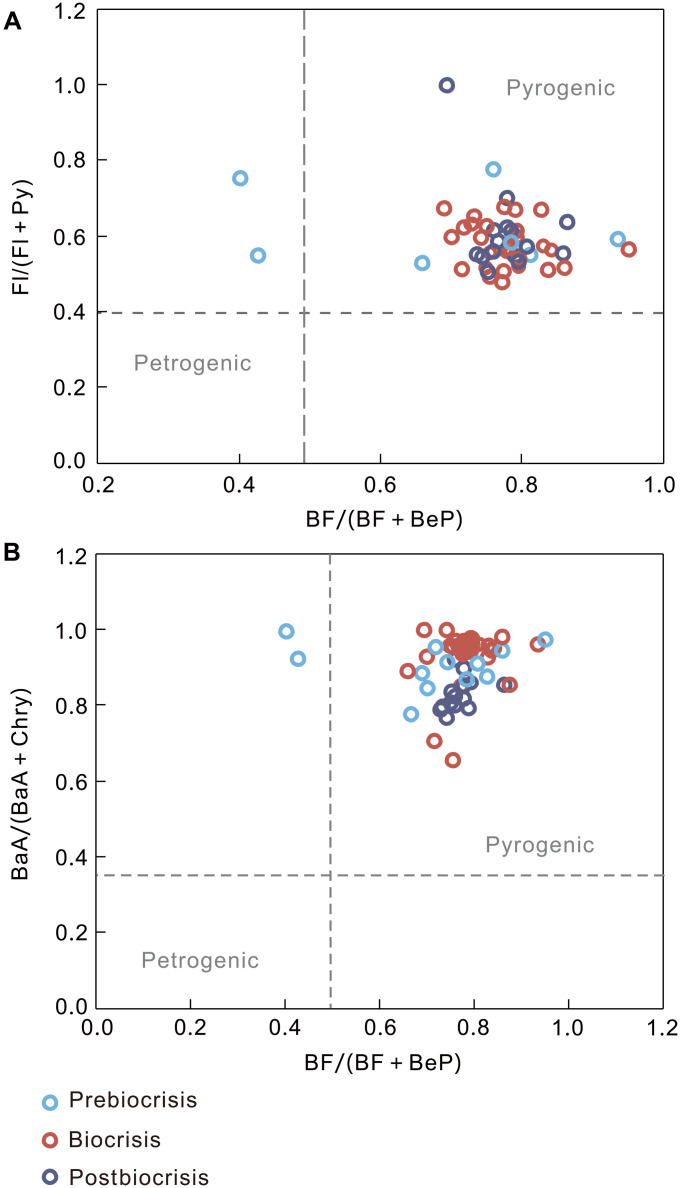
Diagnostic PAHs ratios in the Chattanooga Shale of Chestnut Mound, central Tennessee, United States. Panels (**A**) and (**B**) are cross-plots of diagnostic PAH ratios indicating whether PAHs are of petrogenic or pyrogenic in origin. Fl, fluoranthene; Py, pyrene; BaA, benz[a]anthrene; Chry, chrysene; BF, benzo[b/k/j]fluoranthene; BeP, benzo[e]pyrene.

A range of PAHs were detected in the aromatic fractions of all samples, including fluoranthene (Fl), pyrene (Py), benz[a]anthrene (BaA), chrysene (Chry) ([coeluted with triphenylene (TrP)], benzo[b/k/j]fluoranthene (BF), benzo[a]pyrene (BaP), benzo[e]pyrene (BeP), benzo[g,h,i]perylene (BghiPe), and coronene (Cor) (fig. S1). We examined the distributions of detected PAHs to ensure that the PAH compounds primarily reflect wildfire inputs and were not confounded by modern fossil fuel contamination or derived from geologic sources. A series of diagnostic PAH ratios, i.e., BF/(BF + BeP), Fl/(Fl + Py), and BaA/(BaA + Chry) ratios, were used to assess whether PAHs were derived from pyrogenic or petrogenic sources. Heating processes can occur over millions of years at low temperatures (<150°C), such as during the generation of petroleum (petrogenic), and preferentially produce thermodynamically stable compounds (e.g., Py, BeP, and Chry) (*25*). In contrast, rapid heating at higher temperature (>300°C)—as happens during combustion events such as wildfires, volcanic activity, and asteroid impact (pyrogenic)—tends to produce less stable molecules (e.g., Fl, BF, and BaA) (*25*, *26*). In our samples, all Fl/(Fl + Py) ratios were >0.4, and nearly all samples showed BF/(BF + BeP) > 0.5 and BaA/(BaA + Chry) > 0.35, suggesting the dominance of pyrogenic origin (Fig. 2, A and B). The only exceptions were two samples from the prebiocrisis strata with BF/(BF + BeP) ratios indicative of a petrogenic origin (Fig. 2, A and B).

The additional evidence of wildfires comes from the inertinite macerals, commonly synonymous with charcoals. Inertinite macerals were present throughout the study section (fig. S2). They were predominantly composed of inertodetrinite and semifusinite, which are thought to originate from plant material and commonly attributed to wildfires (*27*–*29*). We also observed macrinite at a minor abundance, which some studies suggest may form through the activity of fungi and bacteria (*30*), although it is more commonly interpreted as a product of strong oxidative reactions typically associated with combustion (*28*, *31*).

Variations in transportation distance and preservation likely affect the abundance of terrigenous organic matter preserved in aquatic sediments (*32*, *33*) and thus the abundance of wildfire compounds and microfossils. To account for the associated biases, we normalized the wildfire markers relative to terrestrial plant markers, i.e., the ratio of pyrogenic PAHs (the sum of Fl, Py, BaA, Chry + TrP, BF, BaP, BeP, BghiPe, and Cor) to C_27_, C_29_, and C_31_ *n*-alkanes [PyC/(PyC + *n*-C_27+29+31_)] and the ratio of inertinites to vitrinite macerals [i.e., In/(In + Vit)] (dataset S1). Long-chain *n*-alkanes (C_27_, C_29_, and C_31_) are mainly derived from leaf waxes of terrestrial land plants (*34*). Their presence in low-maturity marine sedimentary rocks can record vascular plants inputs into aquatic environments (*11*, *35*). Vitrinite macerals are from any tissue with substantial cellulose and lignin (*36*). Similarly, previous studies have used ratios instead of absolute concentrations of combustion products to track wildfires (*28*, *37*). In our samples, PyC/(PyC + *n*-C_27+29+31_) ratios were strongly correlated to In/(In + Vit) ratios (Spearman’s ρ = +0.855, *P* < 0.001), providing further support that these two proxies represent the same process of wildfire activity. Furthermore, although thermal maturity plays an essential role in PAH generation, the relatively low maturity of all samples (*T*_max_ ≤ 438°C) (dataset S1), the high abundance of perylene (fig. S1) (*38*), and the absence of correlations between *T*_max_ versus PyC/(PyC + *n*-C_27+29+31_) (Spearman’s ρ = −0.541, *P* = 0.210) and between *T*_max_ versus In/(In + Vit) ratios (Spearman’s ρ = −0.360, *P* = 0.427) all point to the minimal influence of thermal maturation on the wildfire proxies adopted in this study.

### Wildfire surge during the F-F biotic crisis

**Fig. 3.**
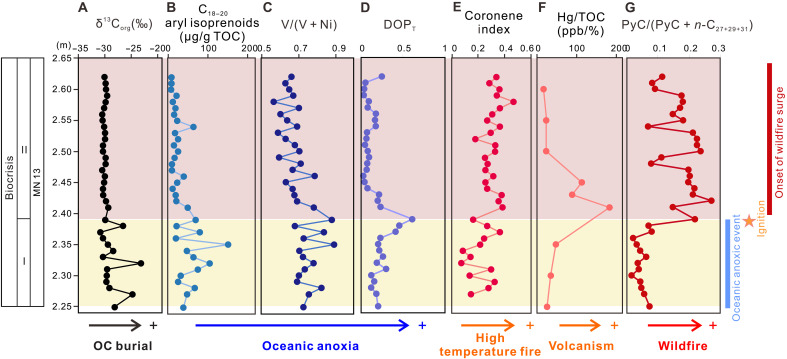
High-resolution geochemical profiles of the F-F biocrisis interval in Chestnut Mound, central Tennessee, United States. (**A**) Stable carbon isotope compositions of OC (δ^13^C_org_), with positive excursions indicate enhanced marine OC burial; (**B** to **D**) C_18–20_ aryl isoprenoids concentrations, V/(V + Ni) ratios and DOP_T_ values, with higher values indicating marine anoxia/euxinia. (**E**) Coronene index, with increase values reflecting high-temperature wildfires. (**F**) Hg contents normalized to TOC (Hg/TOC), with higher values indicating active volcanism. (**G**) Relative concentrations of pyrogenic PAHs [PyC/(PyC + *n*-C_27+29+31_)], with higher values indicating increased wildfires. PyC compounds include Fl, Py, BaA, Chry [coeluted with triphenylene (TrP)], BF, BaP, BeP, benzo[g,h,i]perylene (BghiPe), and coronene (Cor). Horizontal yellow band marks the early stage of the biocrisis event (stage I), and horizontal pink band marks the later stage of the biocrisis event (stage II). ppb, parts per billion.

**Fig. 4.**
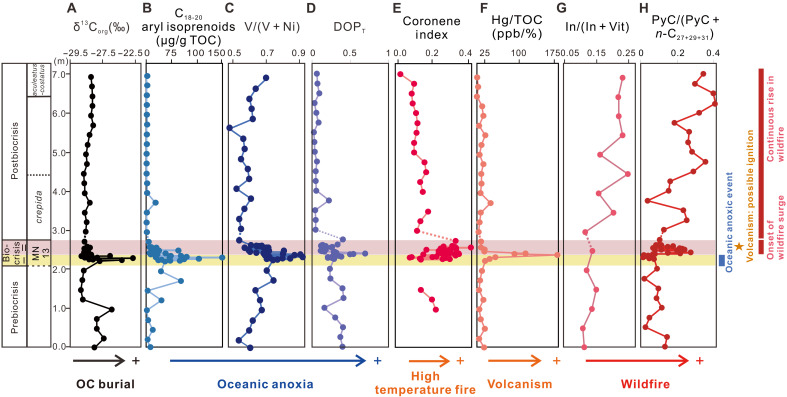
Chemostratigraphic profiles of the 7-m Chattanooga Shale section exposed in Chestnut Mound, central Tennessee, United States. (**A**) Stable carbon isotopic compositions of OC (δ^13^C_org_), with higher values indicating enhanced marine OC burial. (**B** to **D**) Concentrations of C_18–20_ aryl isoprenoids, V/(V + Ni), ratios and DOP_T_ values, with higher values indicating marine anoxia/euxinia. (**E**) Coronene index, with higher values indicating high-temperature wildfires. (**F**) Total Hg contents normalized by TOC (Hg/TOC), with higher values indicating active volcanism. (**G** and **H**) Relative concentrations of inertinite macerals [In/(In + Vit)] and pyrogenic PAHs [PyC/(PyC + *n*-C_27+29+31_)], with higher values indicating increased wildfires. PyC compounds include Fl, Py, BaA, Chry (coeluted with TrP), BF, BaP, BeP, BghiPe, and Cor. Horizontal yellow band marks the early stage of the biocrisis event (stage I), and horizontal pink band marks the later stage of the biocrisis event (stage II). The dash lines in (A) to (G) indicate the possible discontinuous depositions during the Early Famennian.

The F-F extinction event in the Appalachian Basin and adjacent basins has been documented by multiple lines of paleontological evidence, including the abrupt change in the conodont fauna (*39*), the disappearance of shallow-water rugose corals (*40*), the loss of brachiopod diversity (*41*), and reduction in both size and abundance of trace fossils (e.g., burrow and bioturbation) (*42*). The interval examined in the study section was stratigraphically equivalent to the F-F biocrisis intervals in the Appalachian Basin and other well-studied sections worldwide based on a combination of biostratigraphic, sedimentological, and geochemical evidence (details see text S1). Based on the high-resolution data profiles (Fig. 3), the F-F biocrisis interval in our study section can be divided into two stages. Stage I, in the lower 0.14 m of the mass extinction interval, was characterized by peak marine anoxia, as evidenced by positive spikes of δ^13^C_org_, and elevated aryl isoprenoids concentrations, V/(V + Ni) ratios and DOP_T_ values (Fig. 3, A to D, and dataset S1). Positive excursions in δ^13^C_org_ and/or δ^13^C_carb_ have been reported globally from Euramerica, South China, Tarim, and Gondwana (*20*, *43*, *44*), reflecting a globally enhanced burial of OC during the F-F biocrisis interval. Intermediate-chain aryl isoprenoids are commonly used as proxies for photic zone euxinia because they primarily originate from anoxygenic photosynthesis by green sulfur bacteria (Chlorobiaceae) (*45*). Although these compounds can also form during the diagenesis of β-carotene (*46*), the detection of isorenieratane (I), renieratane (R), and paleorenieratane (P) in our samples excludes this pathway and confirms a Chlorobiaceae source. Two additional redox-sensitive proxies were used, V/(V + Ni) (*47*) and DOP_T_ (*48*), and they both showed significant positive correlations with the aryl isoprenoid concentrations [V/(V + Ni): Spearman’s ρ = +0.796, *P* < 0.001); DOP_T_: Spearman’s ρ = +0.647, *P* < 0.001]. Collectively, the coincident increases in the values of δ^13^C_org_, aryl isoprenoids concentrations, V/(V + Ni) ratios, and DOP_T_ indicate intensified marine anoxia enhanced and elevated OC burial (Figs. 3 and 4). Meanwhile, the PyC/(PyC + *n*-C_27+29+31_) ratios fluctuated within a narrow range and yielded similar values to those from the prebiocrisis strata (Figs. 3G and 4H), showing minimal changes in wildfire activity during periods of peak marine anoxia.

The biocrisis stage II, the upper 0.26 m of the F-F interval, showed an opposite pattern. This stage was characterized by a rapid increase in PyC/(PyC + *n*-C_27+29+31_) values, accompanied by declines in δ^13^C_org_, aryl isoprenoids concentrations, V/(V + Ni) ratios, and DOP_T_ (Fig. 3, A to D and G). We therefore defined this stage as the “onset of wildfire surge,” marked by the initial rise in PyC/(PyC + *n*-C_27+29+31_) ratios that indicates that wildfire activity intensified after the peak period of OC burial and marine anoxia. Over longer timescales, wildfire activity remained elevated through the Famennian, as evidenced by sustained increases in PyC/(PyC + *n*-C_27+29+31_) and In/(In + Vit) throughout the postbiocrisis strata (Fig. 4, G and H). In contrast, the postbiocrisis δ^13^C_org_, aryl isoprenoids concentrations, V/(V + Ni) ratios, and DOP_T_ values returned to the prebiocrisis levels and remained low (Fig. 4, A to E), reflecting reduced OC burial and a relatively more oxygenated marine environment following the F-F mass extinction.

Notably, the observation that the increase in wildfires happened subsequent to, rather than before or concurrently with, marine anoxia provides strong evidence that wildfires were a consequence of marine anoxia and intensified OC burial. This interpretation challenges earlier studies, which, due to insufficient temporal resolution, speculated that wildfire contributed to the expansion of marine anoxia and eventually the F-F biotic crisis through mobilizing land-derived nutrients (*10*, *13*, *14*). Rather, our records indicate that marine anoxia and associated OC burial likely promoted subsequent wildfire surges during the Late Devonian.

**Fig. 5.**
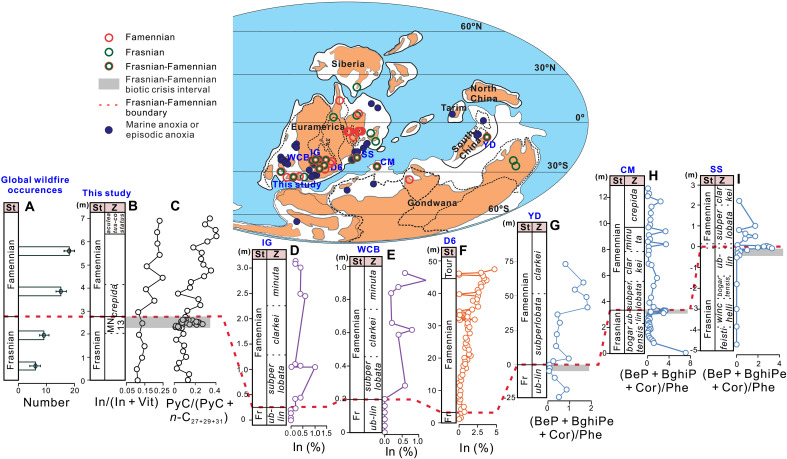
Global distribution and stratigraphic correlation of Late Devonian wildfire proxies. Global paleogeographic map (above) for Late Devonian (370 Ma) [adapted and reprinted from (*99*), copyright 2020, with permission from Elsevier] shows wildfire occurrences and the locality of the study section. The synthetic data of Late Devonian wildfire evidence in (**A**) were from (*12*). Panels (B) to (I) (below) represent continuous stratigraphic variations of wildfire proxies from F-F sections: (**B** and **C**) Chestnut Mound section (this study), (**D**) Irish Gulf (IG) (*101*), (**E**) Walnut Creek Bank (WCB) (*101*), (**F**) D6 core (*10*), (**G**) Yangdi section (YD) (*63*), (**H**) Coumiac section (CM) (*14*), and (**I**) Sinsin section (SS) (*14*). The gray bands in (B) and (G) to (I) mark the F-F biocrisis intervals identified in each section. Specifically, the F-F biocrisis intervals in YD, CM, and SS sections were identified by (*14*, *20*, *102*). St, (sub)stage; Z, conodont zonation; In, inertinite macerals; Vit, vitrinite macerals; Phe, phenanthrene; Fr, Frasnian stage.

Analyzing literature data reveals the global nature of both marine anoxia and wildfire intensification associated with the F-F mass extinction. The global development of oceanic anoxia/euxinia across the F-F boundary is well established, as evidenced by widely distributed depositions of black shale and bituminous limestone near the boundary, along with various geochemical proxies, including redox-sensitive trace metals, pyrite framboids, sulfur isotope, and intermediate-chain aryl isoprenoids (C_18–20_) [e.g., (*49*, *50*)] (see also Fig. 5 for global distribution of marine anoxia/euxinia). In parallel, existing records from Euramerica and South China provide evidence for widespread wildfire intensification after the F-F interval/boundary (Fig. 5). Furthermore, a previous synthesis of Devonian wildfire evidence (fossil charcoals and PAHs) identified a global increase in wildfire occurrences, and this increase occurred after the F-F interval/boundary, a phenomenon that the authors termed the “Famennian Wildfire Explosion (FWE)” (Fig. 5A) (*12*). Our dataset here provides the first, direct evidence capable of resolving the temporal relationship between wildfire activity and OAEs/OC burial, thereby filling a critical gap in our understanding of Late Devonian Earth system dynamics.

### Wildfire surge and *p*O_2_

**Fig. 6.**
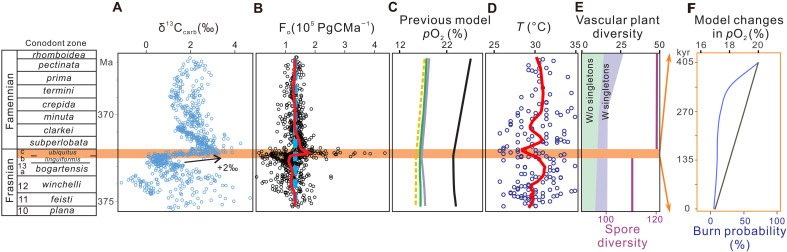
Summary of published data of δ^13^C of carbonate rocks (δ^13^C_carb_) across the F-F boundary. (**A**) Late Frasnian–Early Famennian δ^13^C_carb_ data. (**B**) Calculated OC burial flux (*F*_o_) across the F-F boundary (details of calculation can be found in Materials and Methods). (**C**) Atmospheric oxygen levels (*p*O_2_) across the F-F boundary reconstructed by previous studies [purple line: (*103*); black line: (*21*); green line: (*52*); yellow line: (*28*); the dash line represents the period when no data were reported]. (**D**) Paleotemperature (*T*) records across the F-F boundary calculated by (*104*). (**E**) Species-level vascular plant diversity trends; green and purple shadings outline diversity trend of vascular plant species in Euramerica [modified and reprinted from (*12*), copyright 2021, with permission from Elsevier]. Purple solid line represents spore diversity from (*84*). (**F**) Modeled changes in *p*O_2_ and wildfire probability during the 405 kyr before the F-F boundary. Black line represents *p*O_2_ calculated based on *F*_o_ in this study (see text S2). Blue curve represents burning probability from (*58*). The conodont zonation was from (*97*, *105*). The absolute ages of conodont zone boundaries are from (*106*). The orange band across (A) to (D) outlines the F-F biocrisis interval with a duration of 405 kyr (*20*), which stratigraphically aligns with the *linguiformis* and *ubiquitus* conodont zone (upper part of MN13 zone) (*58*, *106*). Red lines in (B) and (D) represent LOWESS fitting of results. Blue dash line in (B) represents an average value of *F*_o_ during the Late Devonian. Data used to construct these graphs can be found in dataset S2, including δ^13^C_carb_, *F*_o_, their conodont zone and assigned absolute ages. A synthesized *p*O_2_ reconstructed by previous studies, paleotemperature values (*T*), and vascular plant evolutionary patterns throughout the entire Late Devonian were present in fig. S4.

Our data show a temporal delay between the onset of OAEs and the subsequent surge in wildfire activity (Figs. 3 and 4). We hypothesize that this delay reflects the time required for atmospheric oxygen levels to rise sufficiently to sustain widespread combustion. Previous studies demonstrate a sharp increase in *p*O_2_ at the F-F boundary and a continued rise throughout the Famennian [e.g., (*21*, *28*, *51*, *52*)] (Fig. 6C). However, previously modeled *p*O_2_ curves, with a resolution of 10– to 15–million year (Myr) time bins, lack the sufficient temporal resolution needed to explain the wildfire changes observed on the thousand-year (kyr) timescale from the study section. To better constrain oxygen fluctuations at higher temporal resolution, we compiled from previous publications the carbon isotopic records of carbonate (δ^13^C_carb_) of rocks deposited from the earliest Frasnian to the latest Famennian around the world, including 1412 data points from 18 sections across Euramerica, Gondwana and South China epicontinental seas (Fig. 1 and dataset S2). The worldwide records of δ^13^C_carb_ at the F-F boundary document a positive excursion greater than +2‰ (Fig. 6A). Assuming that OC burial increased by the same magnitude as the observed increase in δ^13^C_carb_, we applied a well-established, widely used carbon isotope mass balance model (*53*) to quantify the amount of O_2_ production resulting from OC burial spanning from the earliest Frasnian to the latest Famennian. This model assumed an initial steady-state condition with values representative of the Phanerozoic average carbon inventory (*53*) (summarized in dataset S4), and all subsequent changes were treated as perturbations to the system. Our model results show a dramatic rise in OC burial rate during the latest Frasnian and estimated a rate of up to 2 × 10^5^ Pg per/myr near the F-F boundary (Fig. 6B). For context, this rate was 25% greater than the modern global marine OC burial rate of ~160 TgC/year (*54*). It is widely accepted that OC burial in sediments—ultimately derived by photosynthesis—serves as a net source of atmospheric O_2_ (*55*), and the burial of 1 mol of reduced OC corresponds to the release of 1 mol of O_2_ produced via photosynthesis (*55*, *56*). Hence, this magnitude of burial could allow for 1.67 × 10^13^ mol of oxygen accumulating in Earth’s atmosphere per year during the F-F biocrisis interval.

Assuming that increased OC burial fluxes remained invariant during the F-F biocrisis interval and persisted for a duration of ~405 kyr [i.e., duration of the Upper Kellwasser (UKW)] (*20*), with a dry air reservoir of ~3.8 × 10^19^ mol and a constant oxygen consumption rate of ~3 × 10^12^ mol/year (*56*), the highest OC burial rate would have led to up to 3% rapid rise in *p*O_2_ by the F-F boundary. Wildfire evidence was nearly absent during the Middle Devonian to the Early Frasnian, and this absence has been primarily attributed to low *p*O_2_ (*28*). The observations of wildfire evidence from the prebiocrisis strata suggest that the *p*O_2_ during the late Frasnian should be above 17%, which is considered the minimum threshold necessary for the natural ignition of wildfires (*57*). If the prebiocrisis *p*O_2_ was ~17%, as suggested by most previously established *p*O_2_ curves (Fig. 6C), then there would be a low burning probability (less than 20%) (*58*) and slow response of wildfires as *p*O_2_ would be lower than 19% at the early stage of the F-F biocrisis. However, based on our estimate, the *p*O_2_ could have reached up to 20% across the F-F boundary. This level of *p*O_2_ would be able to lead to a sharp increase in the burning probability (>70%) (*58*) and rapid response of wildfires at the later stage of the F-F mass extinction (Fig. 6F). Similarly, Algeo and Ingall (*51*) reconstructed atmospheric oxygen levels from the Silurian to Carboniferous using sedimentary C_org_/P ratios. They suggested that enhanced OC burial drove a 10% sharp rise in *p*O_2_ levels during the Late Devonian, crossing the combustion threshold and coinciding with the increased abundance of fossil charcoals in the Famennian strata. It also needs to be noted that a synthesis study by Lu *et al.* (*12*) reports only a weak correlation between wildfire occurrences and *p*O_2_ changes across the Devonian. This outcome likely reflects the low temporal resolution of the modeled *p*O_2_ curves they used (one data point per 3.2- to 8.4-Myr time bin), which captured long-term (Myr scale) trends rather than the shorter-term (kyr scale) variations examined in the present study.

Owing to the absence of defined MN13-MN12 conodont boundaries in the study section (*59*), the carbon isotope shifts observed within the study biocrisis interval not only can reflect the UKW alone but also encompass the Lower Kellwasser event (LKW) (see discussions in text S1) (*60*, *61*). Therefore, the study mass extinction interval could have spanned a longer duration (e.g., ~900 Kyr) (*20*), which could result in calculated *p*O_2_ values higher than 20% at the F-F boundary. However, whether the F-F biocrisis interval in the study section contained UKW interval alone or a combination of the UKW and LKW intervals does not affect our conclusion regarding the observed delay in the wildfire surge relative to the onset of OC burial (see detailed explanation in text S1). This delay reflects a process of oxygen accumulation in the atmosphere, coupled with the nonlinear relationship between wildfire probability and *p*O_2_. After the F-F boundary, the OC burial rate stabilized near the background level (i.e., ~1.4 × 10^13^ gC Myr^−1^) (Fig. 6B), which could maintain a constant flux of oxygen into the atmosphere and thereby sustain a rise in wildfire activity throughout the postbiocrisis interval. Notably, similar lags between positive δ^13^C_org_ excursions and subsequent wildfire intensification have been documented during the OAEs of the early Jurassic (*62*) and the Late Cretaceous (*7*), suggesting a comparable mechanism of atmospheric oxygen accumulation and subsequent wildfire responses.

### Ignition source, fuel, and paleoclimate

In addition to atmospheric oxygen, we considered other factors that could affect wildfire frequency and extent, including an ignition source, a suitable climate with low moisture, and the availability of combustible fuel (*63*).

Two primary ignition sources, lightning and volcanism, could have contributed to increased wildfire activity during the F-F period. Lightning is widely considered the dominant natural trigger for paleowildfires (*64*), and today, it is responsible for ~10% of global forest fires (*63*). By the Late Devonian, terrestrial vegetation had developed sufficient height, structural complexity, and areal continuity to serve as effective lightning targets capable of sustaining ignition (*65*). Nevertheless, no direct geological evidence for lightning has yet been identified across the F-F interval, making its contribution difficult to quantify. In contrast, our coronene index and Hg/total organic carbon (TOC) data suggest that volcanism may have served as an additional or alternative ignition source. Several large igneous provinces (LIPs) (e.g., Viluy Traps, Kola, Vyatka, and Pripyat-Dniepr-Deonets rift systems) were active during the F-F period [(*66*) and references therein]. Coronene is a highly condensed PAH that requires higher energy to form relative to PAHs with fewer rings (*67*). Elevated coronene index values (>0.2) have been interpreted to indicate high-temperature organic matter burning (>1200°C) ignited by lava flows, in comparison to normal forest wildfires (<1000°C) (*68*). Coronene enrichment in Late Devonian sections has been previously observed and interpreted as evidence for a strong link between LIP volcanic emissions and the Late Devonian biocrises (*69*). In the present section, elevated Coronene Index values coincided with the initial phase of prolonged wildfires increase (Fig. 3, F and G), suggesting that volcanic activity may have acted as an ignition source. Additional support for this interpretation is provided by the Hg/TOC proxy, which displayed a significant positive correlation with the coronene index (Spearman’s ρ = +0.408, *P* = 0.025) and reached its maximum values at the onset of intensified wildfire activity (Fig. 4F). Higher Hg concentrations and Hg/TOC ratios across Laurussian, Gondwana, and South China continental shelves have similarly been interpreted as evidence for widespread volcanic activities during the F-F period (*69*–*71*). Nevertheless, Hg anomalies in sedimentary successions can also arise from non-LIP processes—such as submarine hydrothermal emissions, enhanced terrestrial input via soil erosion, or Hg sequestration under strongly anoxic/euxinic conditions due to its affinity for sulfides (*72*, *73*). Combined with the relatively low temporal resolution of the Hg/TOC data, these factors suggest that the Hg/TOC evidence should be considered tentative yet supportive. Over the longer timescale, both the coronene index and Hg/TOC declined, while pyrogenic PAHs and inertinite macerals continued to rise after the F-F boundary (Fig. 4, F and H). This decoupling pattern indicates that while volcanism may had triggered the initial wildfires, sustained burning afterward was likely maintained by elevated *p*O_2_ levels rather than continued volcanisms.

Climate can also play a role in the frequency and extent of wildfire (*74*). The F-F paleotemperature record, reconstructed from oxygen isotopes of biogenic apatite in globally distributed sections, shows relatively low values during the late Frasnian, followed by a positive excursion suggesting a global cooling event from the latest Frasnian to earliest Famennian (Fig. 6D) (*75*). A decline in global surface air temperature could have reduced atmospheric water vapor, potentially resulting in increased climate aridity during the latest Frasnian, which, in turn, may have facilitated wildfire surge. However, because this paleotemperature record was reconstructed at Myr scale, it cannot be directly compared with our thousand-year scale wildfire data, leaving the temporal relationship between global cooling and wildfire activity unsolved. A recent study (*76*), using geochemical data collected from Upper Devonian strata in a core from the northern Appalachian Basin, suggests basin freshening and infers a glacio-eustatic fall throughout the F-F interval. However, unlike our wildfire data, which reveal two distinct phases of change within the F-F interval, their dataset does not capture climate variability within the interval, again leaving the timing between global cooling and wildfire changes uncertain. The influence of climate zonation on the spatial distribution of wildfires during the Late Devonian was identified by Lu *et al.* (*77*). By comparing the paleolatitudinal distribution of the Famennian wildfire evidence with the Köppen-Geiger climate classification and the paleo-humidity model of Le Hir *et al.* (*78*), they found that the Famennian wildfire occurrences were concentrated in Euramerica, where arid climatic conditions prevailed during the Late Devonian. In contrast, wildfire evidence was absent in other regions (e.g., South China and Siberia) that experienced humid conditions. This pattern suggests climate was a key factor controlling the spatial variability of wildfire occurrences during the Famennian. However, the potential role of climate change in modulating the temporal variations in wildfire activity across the F-F biocrisis interval was not addressed due to the lack of continuous temporal evidence for climatic aridity. To assess the potential influence of climate aridity on temporal changes in wildfires, we examined the average chain length (ACL) of plant wax *n*-alkanes. Higher ACL values are generally interpreted as indicative of drier climatic conditions [e.g., (*79*)]. In our record, the ACL values decreased during the later stage of the biocrisis event (stage II), followed by a general decline in the Famennian strata (fig. S6), suggesting a trend toward increasing humidity that does not explain the observed temporal increase in wildfire activities. Similar decoupling between wildfire activity and aridity has been reported for other intervals in Earth’s history, such as the Late Cretaceous OAE2 (*7*) and Paleocene-Eocene Thermal Maximum (*33*). Note that the interpretation of ACL remains ambiguous, as it can be influenced by both aridity and vegetation type (*80*–*82*). Moreover, the relationship between ACL and aridity is not always straightforward; some studies have reported absent or even inverse correlations [e.g., (*80*, *82*, *83*)].

Fuel availability—another major control on wildfire activity—is strongly governed by vegetation dynamics. Analyses of vascular plant species and palynological data also reveal a major increase in land plant taxonomic diversity during the Famennian [(*12*, *84*) and references therein] (Fig. 6E), implying a strong association between wildfire activity and land plant evolution. Fossil evidence from Middle Devonian strata in Belgium, Germany, Svalbard, and New York documents the establishment of the earliest forests in central Euramerica before the Late Devonian, dominated by cladoxylopsids, lycopods, and, critically, *Archaeopteris*, which developed advanced, seed plant-like root systems enabling expanded ecological ranges and impacts on terrestrial ecosystems (*85*–*88*). The geographic spread of *Archaeopteris* is further supported by palynological records. Its diagnostic spore, *Geminospora*, has been identified in the Upper Devonian strata from Maryland, Wyoming, West Virginia, and Virginia, extending its distribution across western and south-central Euramerica (*89*, *90*). A recent study (*91*) also demonstrates that *Archaeopetris* became increasingly dominant during and after the F-F biocrisis event in Euramerica, as shown by the rising abundance of *Geminospora* across the δ^13^C_org_ excursions at the F-F boundary in East Greenland. Additional evidence from *Callixylon* wood, trilete spores and plant debris in the Chattanooga Shale, and its stratigraphic equivalents in the southern Appalachian Basin and adjacent basins indicates that *Archaeopteris* forests had expanded into the southern Euramerican landmass, including our study area, by the F-F transition (*11*, *92*, *93*). Lu *et al.* (*12*) shows that the spatial-temporal evolution of *Archaeopteris*-dominated forests coincided with the geographic diversification of Famennian wildfires. The rise of these forests—with their increased tree heights and lignin contents—likely provided a major fuel source for Late Devonian wildfires. Nevertheless, more precise constraints on the timing and pattern of Late Devonian plant diversification are needed to definitively establish this fire-vegetation feedback.

## MATERIALS AND METHODS

### Materials

A total of 65 samples were collected from one publicly accessible field outcrop of the Upper Devonian Chattanooga Shale located in central Tennessee (36.2078°N, 85.834°W), southeastern United States (Fig. 1). The sedimentology and conodont biostratigraphy of Chattanooga Shale in the study section have been described in (*59*) and (*94*). For this study, 30 samples were collected at 25-cm intervals from the Chattanooga Shale of the entire study section, and 35 samples were collected at 1-cm intervals from the F-F biocrisis interval. Before geochemical analyses, fragments of weathered rock surfaces were further removed using knives in the laboratory. Samples were then thoroughly washed using ultrapure carbon-free water, dried in an oven at 40°C, and grounded to 100- to 200-mesh powders. Measurement procedures are described below.

### Bulk organic geochemical analyses

All collected samples were processed for the analyses of TOC and δ^13^C_org_, trace metals, and major oxides. Seven samples at an interval of 1 m were analyzed to determine the thermal maturity parameter of *T*_max_. Thirty-five samples, comprising 10 samples from the biocrisis interval at an interval of 0.02 to 0.05 m and 25 samples from the prebiocrisis and postbiocrisis interval at an interval of 0.25 m, were selected for Hg measurement. Detailed information on the analytical method is presented in text S2.

### Organic petrography

Fifteen samples at an interval of 0.5 m were collected for organic petrographic analyses. About 5 g of each sample was soaked in 10% HCl for 24 hours followed by soaking in 48% HF for 48 hours. Subsequently, the samples were treated with a hot Schultz’s solution (HNO_3_ + KClO_3_) and sodium hydroxide, followed by ultrapure carbon-free water rinse. Dried residues were embedded in epoxy resin, polished, and observed using reflectance microscopy using a Nikon Microphot microscope. The samples were examined under immersion oil through a 40× objective lenses. The abundances of vitrinite and inertinite particles were point-counted (600 points).

### Biomarker analyses

All collected samples were analyzed for molecular biomarkers. Powdered samples were Soxhlet-extracted with a mixture of dichloromethane (DCM)/methanol (MeOH) (97:3, v/v). During the extraction, activated copper was added to the extracts to remove the elemental sulfur. The extracted lipid was subsequently separated into aliphatic, aromatic, and polar fractions via silica gel column chromatography by petroleum ether, benzene, and MeOH, respectively.

The aliphatic fraction was analyzed using an Agilent 7890A gas chromatograph (GC) system [a flame ionization detector (FID)] equipped with a DB-1MS capillary column (60 m by 0.32 mm, 0.25-μm film thickness). The oven temperature was increased from 60° (held for 2 min) to 295°C (held for 30 min) at a rate of 4°C/min. The carrier gas was nitrogen at a flow rate of 1.0 ml/min. The temperature of the inlet and FID was set at 295° and 300°C, respectively. The aromatic hydrocarbons containing PAHs were analyzed on an Agilent 7890B GC-5977A mass spectrometer (MS) equipped with a DB-1MS capillary column (60 m by 0.32 mm, 0.25-μm film thickness). Using helium as the carrier gas (1.0 ml/min), GC oven was heated from 80°C with a 2-min hold, then increased at 3°C/min to 220°C, and lastly at 2°C/min to 300°C with a 30-min hold. The source was operated in 70-eV electron impact mode at 230°C.

Compounds were identified by comparing mass spectra and relative retention time to an external standard composed of 16 PAHs (SV Mix 5, Restek), NIST chemistry webbook, and those reported by previous studies (*4*, *45*). Individual normal alkane (*n*-alkane), PAH compounds, and aryl isoprenoids were quantified by comparing the peak area to that of the internal standards added to the sample prior to the GC and GC-MS measurements [i.e., predeuterated *n-*tetracosane (*n*-C_24_D_50_) for *n*-alkane and predeuterated PAHs (phenanthrene-D_10_ and dibenzothiophene-D_8_) for PAHs]. Based on the concentrations of PAHs, coronene index was calculated as Cor/(BeP + BghiPe + Cor) (*69*). Mass chromatograms of aryl isoprenoids and *n*-alkanes have been published as figures S1 and S2 in (*77*). Measurement procedures of C_40_ carotenoids are described in text S2.

### Quantifying OC burial and oxygen accumulation rate

To better capture the dynamic changes of OC burial across the F-F boundary, we used a simplified, time-dependent, single box model proposed by (*53*) to quantify the continuous, temporal variation in OC burial (*F*_o_) required to explain the observed carbon isotope shifts during the Late Devonian. This simplified equation was expressed as follows (*95*)

$${\partial \delta }^{13}C_{\text{carb}}/\partial t=\left[F_{w}\left(\delta^{13}C_{w}-\delta^{13}C_{\text{carb}}\right)+F_{o}\Delta D\right]/M_{0}$$ (1)

where δ^13^C_carb_ represents stable carbon isotopic composition of Late Devonian carbonates synthesized from previous studies (dataset S2). For a literature search, following key words “Devonian stable carbon isotopic composition”, “δ^13^C” and “carbon isotope” were used in in Google Scholar and the Web of Science. Relevant original research articles were further checked by cross-referencing on the respective journal websites. *t* is the age assigned to each carbon isotope data point, and absolute ages were calculated by using the distance of each data point relative to biostratigraphic zone boundaries given by the authors and the absolute ages given for the biozone boundaries (*57*). The synthesized δ^13^C_carb_ values and their assigned absolute age can be found in dataset S2. In Eq. 1, Δ*D* is isotopic discrimination factor between inorganic and OC (Δ*D* = δ^13^C_carb_ − δ^13^C_org_). In this study, Δ*D* was assumed as 30‰, which is the mean difference between δ^13^C_carb_ and its paired δ^13^C_org_ of the F-F section that were calculated on the basis of synthesized δ^13^C values in (*96*) (dataset S3). As in previous studies (*95*, *97*), we first established the model’s boundary conditions, with the corresponding values (summarized in dataset S4) are representative of high-CO_2_ Phanerozoic carbon inventory (*53*). These values establish a steady state for the exchange of carbon between the oceans and atmosphere before any perturbations during the Late Devonian. *F*_w_ represents input to the ocean combining fluxes delivered from continental weathering and volcanic emission (*95*, *97*). *F*_w_ was assigned a flux of 6 × 10^20^ gC Myr^−1^ with an isotope value (δ^13^*C*_w_) of −5‰. The OC burial flux (*F*_o_) was initially 1.2 × 10^18^ gC Myr^−1^, and carbonate burial flux (*F*_carb_) was initially 4.8 × 10^18^ gC Myr^−1^. *M*_0_ represents the initial concentration of dissolved inorganic carbon in the atmospheric and marine reservoirs and was set to be *M*_0_ = 45.6 × 10^18^ gC with its initial isotope value of zero. These increased input, output, and reservoir values allow the model to begin at a steady state before the perturbations, and δ^13^C_carb_ will change correspondingly to adjust the ocean-atmosphere system to a new steady state. Here, we assumed the only variable that directly and significantly controlled the magnitude of the transient positive excursion was the amount of OC buried. The OC burial rate was calculated for individual section.

To reconstruct a global trend in global OC burial rate during the Late Devonian, we then used Python’s Locally Weighted Scatterplot Smoothing (LOWESS) method and generated a best-fit curve to a total of 1412 data points of calculated OC burial rate. The OC burial in sediments, ultimately derived by photosynthesis, is widely recognized as a net source of atmospheric O_2_ (*55*). Accordingly, we converted OC burial rate into O_2_ source fluxes into atmosphere, following previous studies stating that the burial of 1 mol of reduced OC released 1 mol of O_2_ into atmosphere (*55*, *56*). The accumulation flux of oxygen in the atmosphere was calculated as the source flux of O_2_ into the atmosphere minus the consumption flux. During Earth’s early history, O_2_ consumption was primarily driven by flux of metamorphic and volcanic reductants consumed (*98*), which was set as ~3 × 10^12^ mol/year (*56*).

## Supplementary Materials

### The PDF file includes:

Texts S1 and S2 Figs. S1 to S6 Legends for datasets S1 to S4 References

### Other Supplementary Material for this manuscript includes the following:

Datasets S1 to S4
