# Supplementary material for: Massive wildfires followed oceanic anoxic events during the Late Devonian Frasnian-Famennian mass extinction
Source: Sci Adv. 2026 Apr 1;12(14):eady4534. doi: 10.1126/sciadv.ady4534 (PMC13041755; doi:10.1126/sciadv.ady4534)
Supplement: Supplementary file 1 — Texts S1 and S2 Figs. S1 to S6 Legends for datasets S1 to S4 References [file sciadv.ady4534_sm.pdf]

Science Advances
AAAS

Supplementary Materials for

Massive wildfires followed oceanic anoxic events during the Late Devonian Frasnian-Famennian mass extinction

Man Lu *et al.*

Corresponding author: Man Lu, luman1021@cup.edu.cn; Yuehan Lu, yuehan.lu@ua.edu

Sci. Adv. **12**, eady4534 (2026)
DOI: 10.1126/sciadv.ady4534

The PDF file includes:

Texts S1 and S2
Figs. S1 to S6
Legends for datasets S1 to S4
References

Other Supplementary Material for this manuscript includes the following:

Datasets S1 to S4

Supplementary text 1: Additional Geological Context

The F–F biotic crisis is marked by a succession of extinction pulses and enveloped two distinct latest Frasnian extinction phases, i.e., the Lower Kellwasser (LKW) and the Upper Kellwasser (UKW) events, but the two events cannot be always differentiated (*107*). The conodont biostratigraphy of the Chattanooga Shale in the Chestnut Mound section, though incomplete, has been established, and the F–F boundary was placed at ~2.75 m above the base of the Chattanooga Shale (*59*). Built on the conodont biostratigraphy, the F–F biocrisis interval was identified to be present at 2.25–2.75 m within the MN 13 conodont zone. Previous studies show that the UKW and LKW intervals occurred within the latest Frasnian upper MN 13 zone (*linguiformis* and *ubiquitus* conodont zone, formerly upper *linguiformis* conodont zone) and MN 12–13 zone boundary (*bogartensis-winchelli* conodont zone, formally lower *rhenana* conodont zone) (*106, 108*), respectively. The upper part of the MN 13 zone is equivalent to the *linguiformis* and *ubiquitus* conodont zones (upper *linguiformis* conodont zone in the older conodont zonation) (*106, 108*), with the upper boundary of the MN 13 conodont zone. Correlation between old and new conodont zonation is shown in Supplementary fig. S4 (sourced is from (*100*)). The presence of the latest Frasnian conodonts (i.e., *Palmatolepis linguiformis*) (*59*) and a volcanic ash bed (i.e., the Center Hill Ash) (*109*) indicates that the study section contained the latest Frasnian rocks and upper part of the MN 13 zone. Also, a positive excursion of $\delta^{13}C_{org}$ and a spike of C_{28}/C_{29} sterane ratios (see Fig. 1 in Lu et al. (*77*)) was observed just below the F–F boundary. These patterns have been identified for the UKW interval in globally distributed sections (*20, 110*). Lu et al. (*77*) correlated the high-resolution XRF-derived Ti/Al records of the study section to other detrital input proxy series (Ti/Al ratios,

magnetic susceptibilities) of the F–F boundary sections distributed around the world (Fig. 7 in (*60, 77*) and Supplementary fig. S5). Based on the variation patterns of the detrital proxies and previously established astronomical timescale for the study section, Lu et al. (*77*) suggests that the UKW and LKW intervals occurred between 2.2–2.75 m (i.e., F–F mass extinction interval in the present study) and 0.6–0.9 m in the study section, respectively. Episodic deposition and disconformities have been documented in the Chattanooga Shale and its stratigraphic equivalents across the Appalachian Basin (*60, 61*). Because conodont data from our section did not allow precisely constrain the boundary between the MN 13 and MN 12 zones, the LKW event could not be robustly identified. As a result, the F–F biocrisis interval recognized at 2.25–2.75 m in our section may also include the LKW interval. Whether this interval represented only the UKW interval or a combination of the UKW and LKW intervals does not affect our key observations that the increase in wildfire proxies followed the positive excursion in $\delta^{13}C_{org}$. In addition, an unconformity has been reported in the early Famennian strata of the central Appalachian Basin (*60, 61*). Owing to the absence of diagnostic early Famennian conodont zones (*subperlobata*, *clarkei* and *minuta*), it is possible that the earliest Famennian rocks were missing from our section. This gap does not affect the overall temporal trends of $\delta^{13}C_{org}$ and marine anoxia proxies, which had already returned to pre-biocrisis values prior to the F–F boundary (Figs. 3 and 4). However, the timing of the onset of the Famennian wildfires may be influenced—occurring either during or immediately after the potentially missing interval. Regardless, the sequence of events remains consistent: wildfire activity followed marine anoxia. The only potential difference lies in the duration of the lag between the anoxia event and the rise in wildfire activity, which may have been longer than currently observed. Therefore, the

possible stratigraphic gap does not affect our central conclusion that the wildfire surge postdated the onset of organic carbon burial.

Supplementary text 2: Methods

Provenance

All rock materials analyzed in this study were collected from the Chattanooga Shale of the Chestnut Mound outcrop, located in central Tennessee, USA, during field trips conducted in 2018. The specimens were collected by Molecular Eco-Geochemistry (MEG) research group, University of Alabama, following standard field and sampling protocols. The authenticity and stratigraphic integrity of the rock materials were validated through detailed sedimentological and stratigraphic correlation of the sampled horizons. The ages of the collected strata were constrained using biostratigraphy and chemostratigraphy, as described in the Methods section and Supplementary text 1, and were conducted by MEG research group. All samples analyzed in this study are permanently curated at MEG lab at Department of Geological Sciences, University of Alabama. The materials are accessible to qualified researchers upon reasonable request in accordance with the policies of the hosting institution.

Bulk TOC, $\delta^{13}C_{org}$ analyses and T_{max}

The powdered samples were treated with 5% sulfurous acid for 24 h to remove carbonate, rinsed with ultra-pure carbon-free water to a neutral pH and then oven-dried at 50 °C for 72 h. Sedimentary TOC and $\delta^{13}C_{org}$ were measured using a Micro Cube elemental analyzer (Elementar Analysensysteme GmbH, Hanau, Germany) interfaced to a PDZ Europa 20–20 isotope ratio mass spectrometer (Sercon Ltd., Cheshire, UK). The isotopic ratios were reported relative to the V-PDB standard with a precision of ±0.2‰ or better.

Seven samples were submitted to the Source Rock Lab, GeoMark Research Inc (Humble, TX, USA) to determine the thermal maturity parameter of T_{max}. T_{max} values were determined by using a HAWK Pyrolysis instrument. The temperature program began with a 300 °C isotherm for 3 min, and then increased to 650 °C at a rate of 25 °C/min.

Shale element analyses

Concentrations of trace metals, major oxides, and total Hg were measured at the ALS Chemex Lab, Ltd (Reno, NV, USA). For trace element analyses, 2–5 mg of grounded sample was treated using a three-acid (HF-HNO_3-$HClO_4$) digestion method. The residue was then leached with HCl. The analysis of the solution was conducted using a PerkinElmer Elan9000 element inductively coupled plasma mass spectrometer (ICP-MS). Analytical precisions for all trace metals were better than 5%. For trace Hg content, powders were tested following the method of Hg-MS42, which used low-temperature aqua regia digestion prior to inductively coupled plasma mass spectrometry (ICP-MS) analysis with an analytical precision better than 7%.

C_{40} carotenoids analyses

Non-polar fractions of selected samples were analyzed using an Agilent 7890B gas chromatography (GC) coupled to an Agilent 7010A triple quadrupole mass spectrometer (GC-MS/MS) operated in multiple reactions monitoring (MRM) modes. The GC was equipped with a DB-5MS column (60 m × 250 μm× 0.25 μm). Samples were introduced via a multimode injector at an initial temperature at 60 °C, which was ramped to 340 °C at a rate of 700 °C/min. The GC oven was held at 60 °C for 2 min, increased to 220 °C at a rate of 8 °C/min followed by a ramp of 2 °C/min to 325 °C, then held isothermally for 35.5 min. The column flow was

set to 1.5 ml/min. The source and transfer line temperatures were set to 280 °C and 320 °C, respectively. The electron energy was 50 eV. Samples were run under multiple reactions monitoring (MRM) mode using paired precursor-product ions optimized for each compound. The compounds were identified using MassHunter Qualitative software based on their full scan spectra and relative retention times in comparison with previously reported chromatograms (*111–113*).

Supplementary figures:

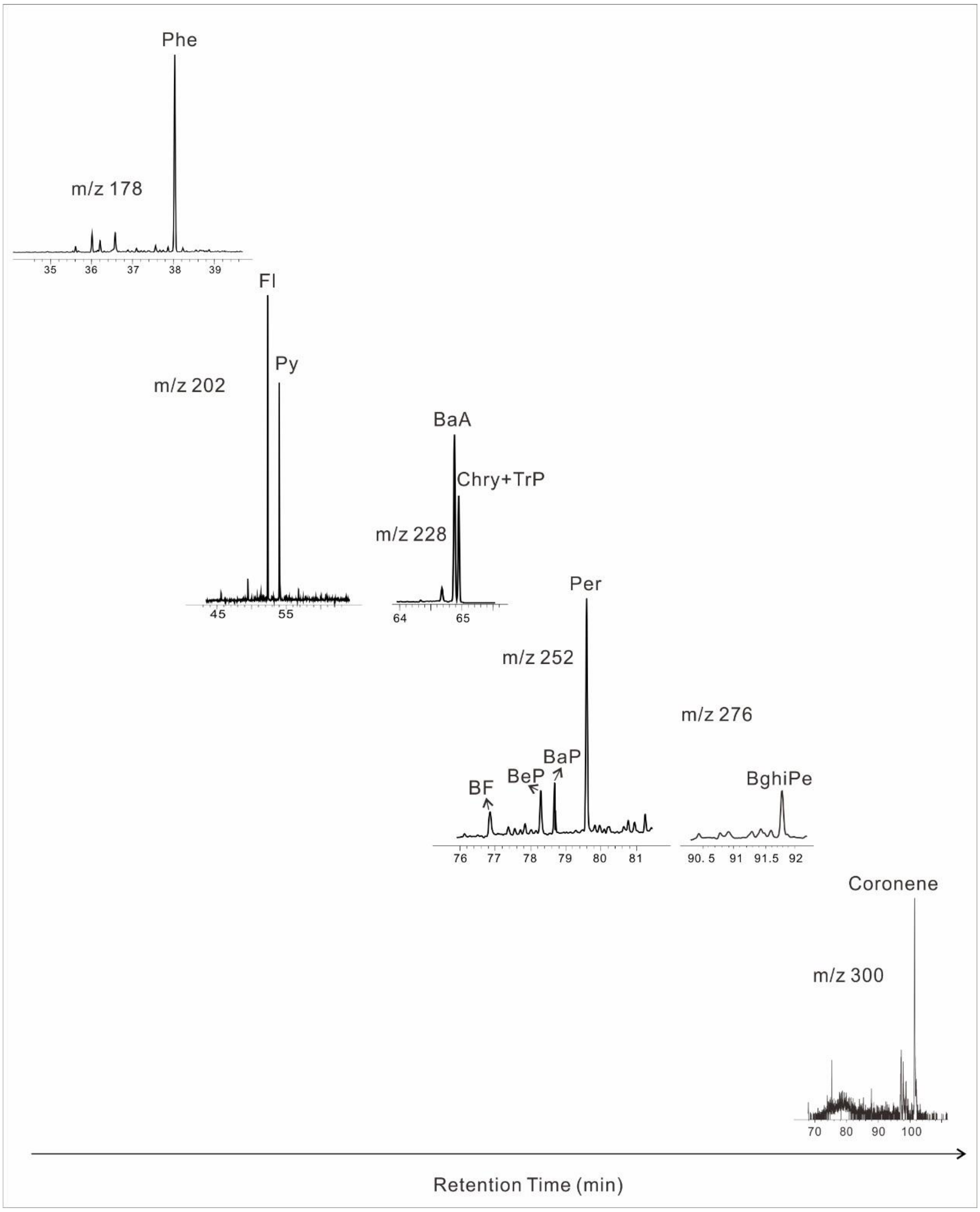

fig. S1. Chromatogram of polycyclic aromatic hydrocarbons (PAHs) in one representative sample of Chattanooga Shale collected from Chestnut Mound, central Tennessee. Phe: phenanthrene; Fl: fluoranthene; Py: pyrene; BaA: benz[a]anthrene; Chry: chrysene (co-eluted with triphenylene (TrP)); BF: benzo[b/k/j]fluoranthene; BaP: benzo[a]pyrene; BeP: benzo[e]pyrene; BghiPe: benzo[g,h,i]perylene; Cor: coronene.

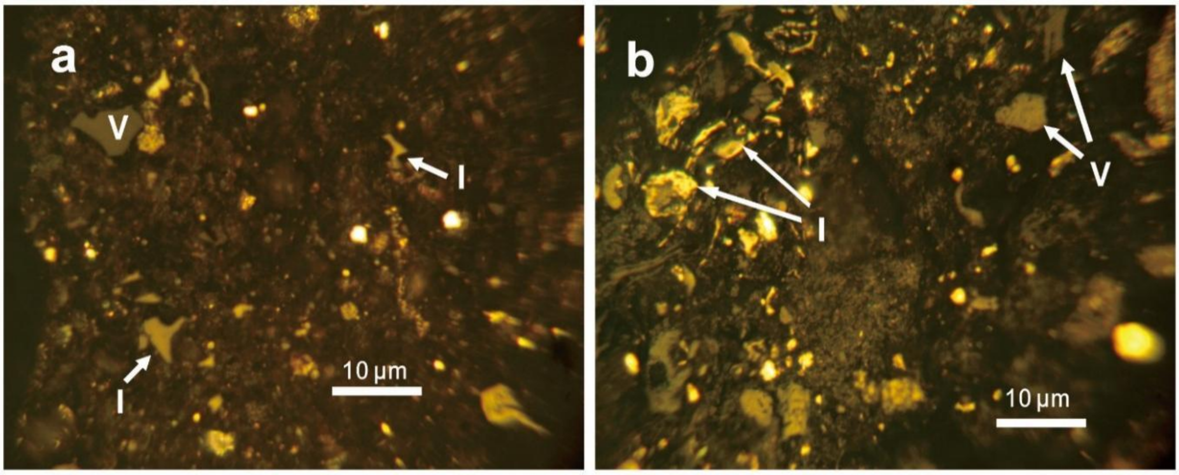

fig. S2. Photomicrographs (white reflected light, oil immersion) of representative samples from (a) Frasnian interval and (b) Famennian interval of the Chattanooga Shale at Chestnut Mound, central Tennessee, USA. V=Vitrinite; I=Inertinite.

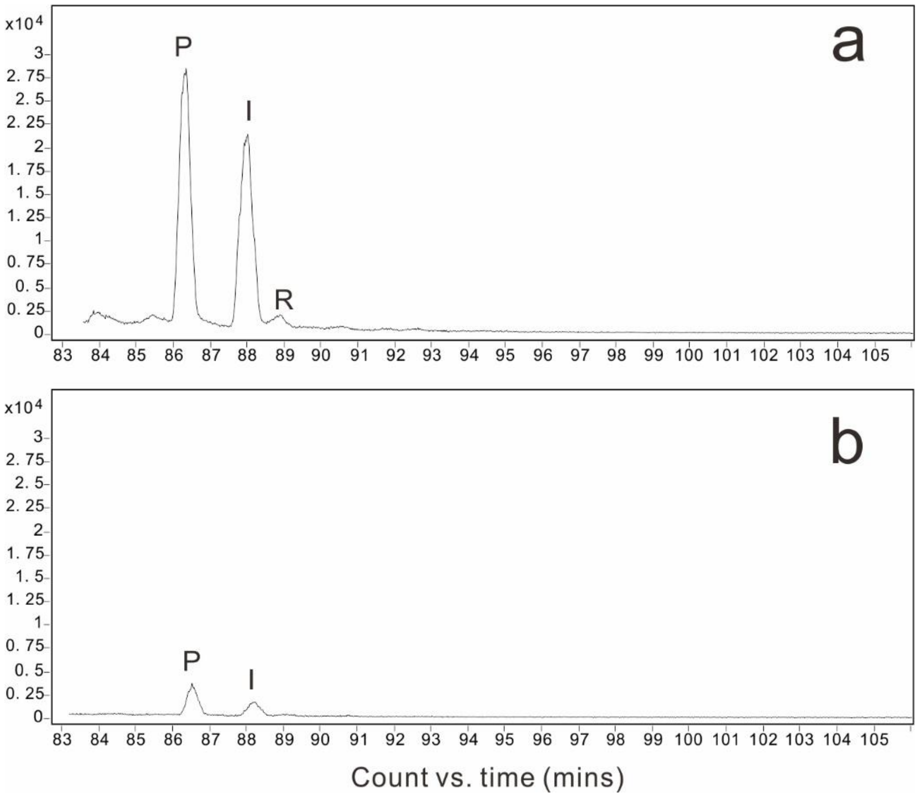

fig. S3. Multiple reaction monitoring chromatograms (m/z 546→134) showing the carotenoids distribution in (a) a Frasnian sample and (b) a Famennian sample of the Chattanooga Shale in Chestnut Mound, central Tennessee, USA. P: paleorenieratane; I: isorenieratane; R: renieratane.

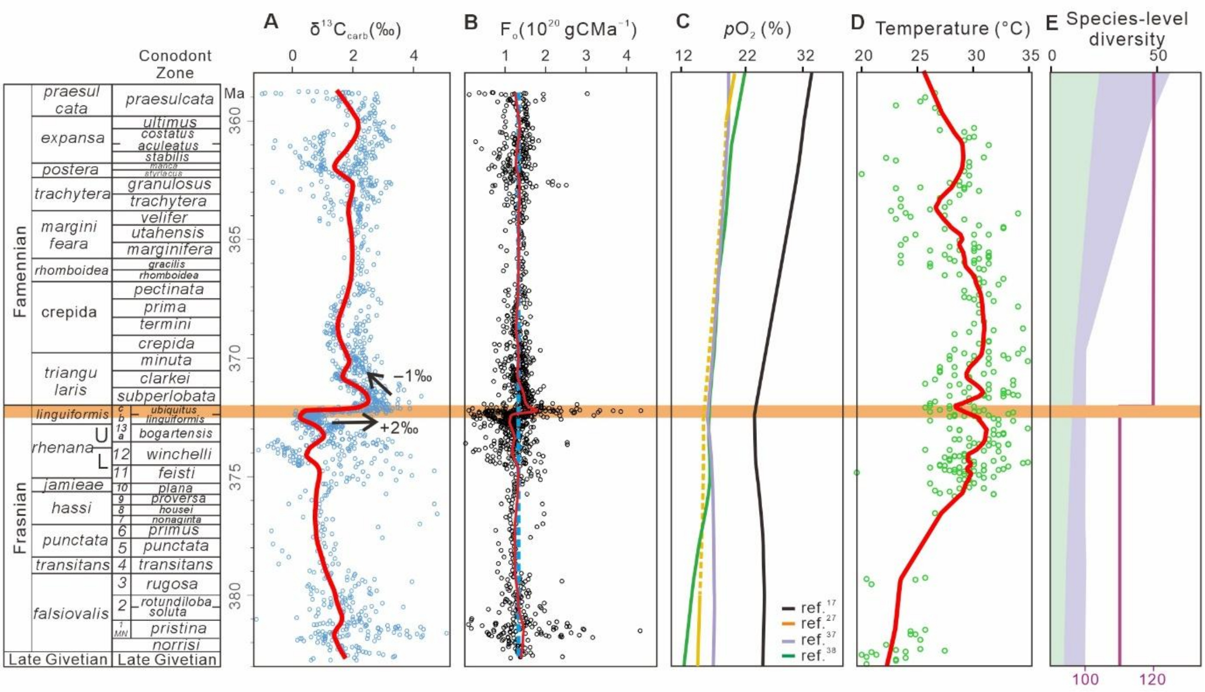

fig. S4. Late Devonian global records of carbonate $\delta^{13}C$, organic carbon burial flux (F_o), atmospheric oxygen level (pO_2), paleotemperature (T), and vascular plant evolutionary pattern. Red lines in panels (b) and (c) represent LOWESS fitting of results. Blue dash line in panel (b) represents average F_o during the Late Devonian. (j) shows Late Devonian pO_2 level reconstructed by previous studies (purple line: (*103*); black line: (*21*); green line: (*52*); yellow line: (*28*)) (d) paleotempeture records (T) from (*104*). (e) green and purple shadings outline species-level diversity trends of vascular plant species in Euramerica from (*12*). The purple solid line represents spore diversity from (*84*) Correlation between old and new conodont zonation is from (*100*). The absolute ages of conodont zone boundaries are from (*106*). The orange band in panels (a–e) represents the Upper Kellwasser (UKW) interval at global scale.

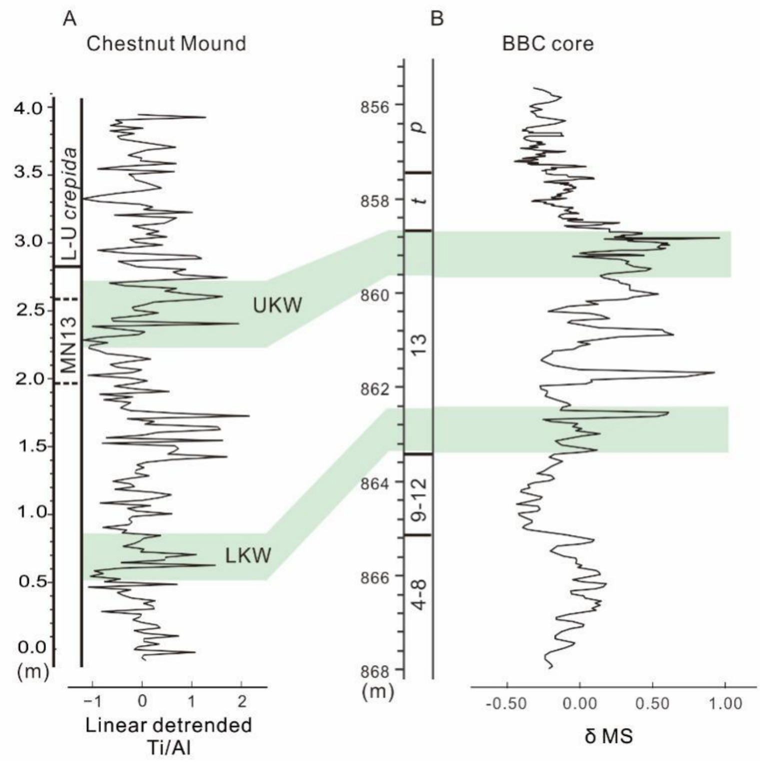

fig. S5. Correlations between detrital proxies of the Chattanooga Shale in study section (Chestnut Mound, panel A) and BBC core in Illinois Basin in (*60*) (panel B). MS: magnetic susceptibility. UKW: Upper Kellwasser interval. LKW: Lower Kellwasser interval.

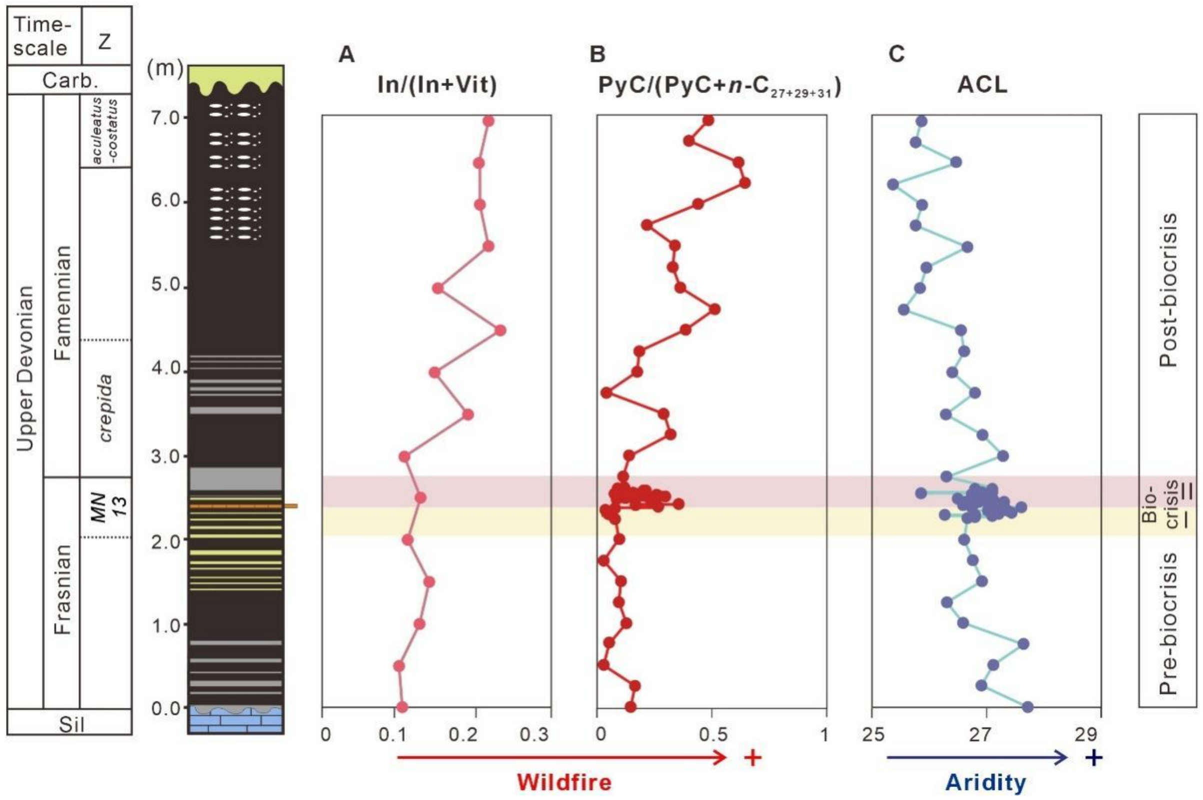

fig. S6. Stratigraphic variations in PyC/(PyC+n-C$_{27+29+31}$), In/(In+Vit) and ACL of the Chattanooga Shale in Chestnut Mound, central Tennessee, USA. PyC: pyrogenic PAHs including fluoranthene (Fl), pyrene (Py), benz[a]anthrene (BaA), chrysene (Chry) (co-eluted with triphenylene (TrP)), benzo[b/k/j]fluoranthene (BF), benzo[a]pyrene (BaP), benzo[e]pyrene (BeP), benzo[g,h,i]perylene (BghiPe) and coronene (Cor). In: inertinite macerals. Vit: vitrinite macerals. Phe: phenanthrene. ACL: average chain length of plant wax n-alkanes.

Captions for supplementary data:

Supplementary Dataset 1. Geochemical data for the Chattanooga Shale in Chestnut Mound, central Tennessee, USA.

Supplementary Dataset 2. Carbon isotopic composition of the Late Devonian carbonates ($\delta^{13}C_{carb}$) synthesized from previous studies, with corresponding conodont zones, sampling heights, assigned absolute ages and calculated organic carbon influx (F_o).

Supplementary Dataset 3. Calculated isotopic discrimination factor between inorganic and organic carbon (ΔD) for Devonian rocks.

Supplementary Dataset 4. Late Devonian relevant values used in the carbon isotope model and carbon mass balance equation.

REFERENCES

1. C.M. Belcher, *Fire phenomena and the Earth system: An interdisciplinary guide to fire science* (John Wiley & Sons, 2013).

2. M. A. Moritz, E. Batllori, R. A. Bradstock, A. M. Gill, J. Handmer, P. F. Hessburg, J. Leonard, S. McCaffrey, D. C. Odion, T. Schoennagel, Learning to coexist with wildfire. *Nature* **515**, 58–66 (2014).

3. W. Shen, Y. Sun, Y. Lin, D. Liu, P. Chai, Evidence for wildfire in the Meishan section and implications for Permian–Triassic events. *Geochim. Cosmochim. Acta* **75**, 1992–2006 (2011).

4. L. Marynowski, B. R. Simoneit, Widespread Upper Triassic to Lower Jurassic wildfire records from Poland: Evidence from charcoal and pyrolytic polycyclic aromatic hydrocarbons. *Palaios* **24**, 785–798 (2009).

5. C. M. Belcher, L. Mander, G. Rein, F. X. Jervis, M. Haworth, S. P. Hesselbo, I. J. Glasspool, J. C. McElwain, Increased fire activity at the Triassic/Jurassic boundary in Greenland due to climate-driven floral change. *Nat. Geosci.* **3**, 426–429 (2010).

6. S. P. Hesselbo, D. R. Gröcke, H. C. Jenkyns, C. J. Bjerrum, P. Farrimond, H. S. Morgans Bell, O. R. Green, Massive dissociation of gas hydrate during a Jurassic oceanic anoxic event. *Nature* **406**, 392–395 (2000).

7. F. G. Boudinot, J. Sepúlveda, Marine organic carbon burial increased forest fire frequency during Oceanic Anoxic Event 2. *Nat. Geosci.* **13**, 693–698 (2020).

8. I. J. Glasspool, R. A. Gastaldo, Silurian wildfire proxies and atmospheric oxygen. *Geology* **50**, 1048–1052 (2022).

9. L. Marynowski, M. Zatoń, M. Rakociński, P. Filipiak, S. Kurkiewicz, T. J. Pearce, Deciphering the upper Famennian Hangenberg Black Shale depositional environments based on multi-proxy record. *Palaeogeogr. Palaeoclimatol. Palaeoecol.* **346**, 66–86 (2012).

10. S. M. Rimmer, S. J. Hawkins, A. C. Scott, W. L. Cressler, The rise of fire: Fossil charcoal in late Devonian marine shales as an indicator of expanding terrestrial ecosystems, fire, and atmospheric change. *Am. J. Sci.* **315**, 713–733 (2015).

11. M. Lu, Y. Lu, T. Ikejiri, N. Hogancamp, Y. Sun, Q. Wu, R. Carroll, I. Çemen, J. Pashin, Geochemical evidence of first forestation in the southernmost Euramerica from Upper Devonian (Famennian) black shales. *Sci. Rep.* **9**, 7581 (2019).

12. M. Lu, T. Ikejiri, Y. Lu, A synthesis of the Devonian wildfire record: Implications for paleogeography, fossil flora, and paleoclimate. *Palaeogeogr. Palaeoclimatol. Palaeoecol.* **571**, 110321 (2021).

13. Z. Liu, H. Tian, D. Selby, J. Hu, D. J. Over, Organic geochemistry evidence for wildfire and elevated pO_2 at the Frasnian–Famennian boundary. *Glob. Planet. Change* **216**, 103904 (2022).

14. K. Kaiho, S. Yatsu, M. Oba, P. Gorjan, J. G. Casier, M. Ikeda, A forest fire and soil erosion event during the Late Devonian mass extinction. *Palaeogeogr. Palaeoclimatol. Palaeoecol.* **392**, 272–280 (2013).

15. D. Bond, P. B. Wignall, G. Racki, Extent and duration of marine anoxia during the Frasnian–Famennian (Late Devonian) mass extinction in Poland, Germany, Austria and France. *Geol. Mag.* **141**, 173–193 (2004).

16. T. J. Algeo, J. Shen, Theory and classification of mass extinction causation. *Natl. Sci. Rev.* **11**, nwad237 (2024).

17. W. Buggisch, The global Frasnian-Famennian »Kellwasser Event«. *Geol. Rundsch.* **80**, 49–72 (1991).

18. M. Gereke, E. Schindler, "Time-specific facies" and biological crises—The Kellwasser event interval near the Frasnian/Famennian boundary (Late Devonian). *Palaeogeogr. Palaeoclimatol. Palaeoecol.* **367–368**, 19–29 (2012).

19. M. Joachimski, R. Pancost, K. Freeman, C. Ostertag-Henning, W. Buggisch, Carbon isotope geochemistry of the Frasnian–Famennian transition. *Palaeogeogr. Palaeoclimatol. Palaeoecol.* **181**, 91–109 (2002).

20. D. De Vleeschouwer, A. C. Da Silva, M. Sinnesael, D. Chen, J. E. Day, M. T. Whalen, Z. Guo, P. Claeys, Timing and pacing of the Late Devonian mass extinction event regulated by eccentricity and obliquity. *Nat. Commun.* **8**, 2268 (2017).

21. R. A. Berner, Phanerozoic atmospheric oxygen: New results using the GEOCARBSULF model. *Am. J. Sci.* **309**, 603–606 (2009).

22. T.M. Lenton, "Fire phenomena and the Earth system: An interdisciplinary guide to fire science," in *Fire feedbacks on atmospheric oxygen*, C. M. Belcher Ed. (John Wiley & Sons, 2013), pp. 289–308.

23. J. Schieber, Evidence for high-energy events and shallow-water deposition in the Chattanooga Shale, Devonian, central Tennessee, USA. *Sediment. Geol.* **93**, 193–208 (1994).

24. G. R. McGhee Jr., P. M. Sheehan, D. J. Bottjer, M. L. Droser, Ecological ranking of Phanerozoic biodiversity crises: Ecological and taxonomic severities are decoupled. *Palaeogeogr. Palaeoclimatol. Palaeoecol.* **211**, 289–297 (2004).

25. E. Stogiannidis, R. Laane, Source characterization of polycyclic aromatic hydrocarbons by using their molecular indices: An overview of possibilities. *Rev. Environ. Contam. Toxicol.* **234**, 49–133 (2015).

26. Z. Wang, M. Fingas, Y. Shu, L. Sigouin, M. Landriault, P. Lambert, R. Turpin, P. Campagna, J. Mullin, Quantitative characterization of PAHs in burn residue and soot samples and differentiation of pyrogenic PAHs from petrogenic PAHs—The 1994 mobile burn study. *Environ. Sci. Technol.* **33**, 3100–3109 (1999).

27. I. J. Glasspool, Palaeoecology of selected South African export coals from the Vryheid Formation, with emphasis on the role of heterosporous lycopods and wildfire derived inertinite. *Fuel* **82**, 959–970 (2003).

28. I. J. Glasspool, A. C. Scott, Phanerozoic concentrations of atmospheric oxygen reconstructed from sedimentary charcoal. *Nat. Geosci.* **3**, 627–630 (2010).

29. D. Uhl, A. Jasper, Wildfire during deposition of the "Illinger Flözzone" (Heusweiler-Formation, "Stephanian B", Kasimovian–Ghzelian) in the Saar-Nahe Basin (SW-Germany). *Palaeobiodiversity Palaeoenvironments* **101**, 9–18 (2021).

30. J. C. Hower, J. M. O'Keefe, C. F. Eble, A. Raymond, B. Valentim, T. J. Volk, A. R. Richardson, A. B. Satterwhite, R. S. Hatch, J. Stucker, Notes on the origin of inertinite macerals in coal: Evidence for fungal and arthropod transformations of degraded macerals. *Int. J. Coal Geol.* **86**, 231–240 (2011).

31. C. Scott, I. J. Glasspool, Observations and experiments on the origin and formation of inertinite group macerals. *Int. J. Coal Geol.* **70**, 53–66 (2007).

32. M.A. Sephton, R.J. Veefkind, C.V. Looy, H. Visscher, H. Brinkhuis, J.W. de Leeuw, "Lateral variations in end-Permian organic matter," in *Geological and Biological Effects of Impact Events*, E. Buffetaut, C. Koeberl, Eds. (Springer, 2001), pp. 11–24.

33. E. H. Denis, N. Pedentchouk, S. Schouten, M. Pagani, K. H. Freeman, Fire and ecosystem change in the Arctic across the Paleocene–Eocene Thermal Maximum. *Earth Planet. Sci. Lett.* **467**, 149–156 (2017).

34. G. Eglinton, R. J. Hamilton, Leaf Epicuticular Waxes: The waxy outer surfaces of most plants display a wide diversity of fine structure and chemical constituents. *Science* **156**, 1322–1335 (1967).

35. K. L. Kennedy, M. R. Gibling, C. F. Eble, R. A. Gastaldo, P. G. Gensel, U. Werner-Zwanziger, R. A. Wilson, Lower Devonian coaly shales of northern New Brunswick, Canada: Plant accumulations in the early stages of terrestrial colonization. *J. Sediment. Res.* **83**, 1202–1215 (2013).

36. International Committee for Coal and Organic Petrology (ICCP), The new vitrinite classification (ICCP System 1994). *Fuel* **77**, 349–358 (1998).

37. T. Karp, A. K. Behrensmeyer, K. H. Freeman, Grassland fire ecology has roots in the late Miocene. *Proc. Natl. Acad. Sci. U.S.A.* **115**, 12130–12135 (2018).

38. L. Marynowski, J. Smolarek, Y. Hautevelle, Perylene degradation during gradual onset of organic matter maturation. *Int. J. Coal Geol.* **139**, 17–25 (2015).

39. D. J. Over, Conodont biostratigraphy of the Java Formation (Upper Devonian) and the Frasnian–Famennian boundary in western New York State. *Geol. Soc. Am. Spec. Paper* **321**, 161–177 (1997).

40. M. Bush, J. D. Csonka, G. V. DiRenzo, D. J. Over, J. A. Beard, Revised correlation of the Frasnian–Famennian boundary and Kellwasser Events (Upper Devonian) in shallow marine paleoenvironments of New York State. *Palaeogeogr. Palaeoclimatol. Palaeoecol.* **433**, 233–246 (2015).

41. J. Day, B.J. Witzke, "Upper Devonian biostratigraphy, event stratigraphy, and Late Frasnian Kellwasser extinction bioevents in the Iowa Basin: Western Euramerica," in *Stratigraphy & Timescales* (Elsevier, 2017), vol. 2, pp. 243–332.

42. D. L. Boyer, E. E. Haddad, E. S. Seeger, The last gasp: Trace fossils track deoxygenation leading into the Frasnian–Famennian extinction event. *Palaios* **29**, 646–651 (2014).

43. P. E. Playford, D. J. McLaren, C. J. Orth, J. S. Gilmore, W. D. Goodfellow, Iridium anomaly in the Upper Devonian of the Canning Basin, western Australia. *Science* **226**, 437–439 (1984).

44. T. J. Suttner, E. Kido, X. Chen, R. Mawson, J. A. Waters, J. Frýda, D. Mathieson, P. D. Molloy, J. Pickett, G. D. Webster, B. Frýdová, Stratigraphy and facies development of the marine Late Devonian near the Boulongour Reservoir, northwest Xinjiang, China. *J. Asian Earth Sci.* **80**, 101–118 (2014).

45. K. Grice, C. Cao, G. D. Love, M. E. Böttcher, R. J. Twitchett, E. Grosjean, R. E. Summons, S. C. Turgeon, W. Dunning, Y. Jin, Photic zone euxinia during the Permian-Triassic superanoxic event. *Science* **307**, 706–709 (2005).

46. M. P. Koopmans, S. Schouten, M. E. Kohnen, J. S. Sinninghe Damsté, Restricted utility of aryl isoprenoids as indicators for photic zone anoxia. *Geochim. Cosmochim. Acta* **60**, 4873–4876 (1996).

47. L. Riquier, N. Tribovillard, O. Averbuch, X. Devleeschouwer, A. Riboulleau, The Late Frasnian Kellwasser horizons of the Harz Mountains (Germany): Two oxygen-deficient periods resulting from different mechanisms. *Chem. Geol.* **233**, 137–155 (2006).

48. T. J. Algeo, J. Liu, A re-assessment of elemental proxies for paleoredox analysis. *Chem. Geol.* **540**, 119549 (2020).

49. E. E. Haddad, M. L. Tuite, A. M. Martinez, K. Williford, D. L. Boyer, M. L. Droser, G. D. Love, Lipid biomarker stratigraphic records through the Late Devonian Frasnian/Famennian boundary: Comparison of high- and low-latitude epicontinental marine settings. *Org. Geochem.* **98**, 38–53 (2016).

50. A. Riboulleau, A. Spina, M. Vecoli, L. Riquier, M. Quijada, N. Tribovillard, O. Averbuch, Organic matter deposition in the Ghadames Basin (Libya) during the Late Devonian—A multidisciplinary approach. *Palaeogeogr. Palaeoclimatol. Palaeoecol.* **497**, 37–51 (2018).

51. T. J. Algeo, E. Ingall, Sedimentary C_{org}: P ratios, paleocean ventilation, and Phanerozoic atmospheric pO_2. *Palaeogeogr. Palaeoclimatol. Palaeoecol.* **256**, 130–155 (2007).

52. B. J. Mills, A. J. Krause, I. Jarvis, B. D. Cramer, Evolution of atmospheric O_2 through the Phanerozoic, revisited. *Annu. Rev. Earth Planet. Sci.* **51**, 253–276 (2023).

53. L. R. Kump, M. A. Arthur, Interpreting carbon-isotope excursions: Carbonates and organic matter. *Chem. Geol.* **161**, 181–198 (1999).

54. D. J. Burdige, Preservation of organic matter in marine sediments: Controls, mechanisms, and an imbalance in sediment organic carbon budgets? *Chem. Rev.* **107**, 467–485 (2007).

55. R. A. Berner, Burial of organic carbon and pyrite sulfur in the modern ocean: Its geochemical and environmental significance. *Am. J. Sci.* **282**, 451–473 (1982).

56. D. C. Catling, M. W. Claire, How Earth's atmosphere evolved to an oxic state: A status report. *Earth Planet. Sci. Lett.* **237**, 1–20 (2005).

57. T. M. Lenton, A. J. Watson, Redfield revisited: II. What regulates the oxygen content of the atmosphere? *Global Biogeochem. Cycles* **14**, 249–268 (2000).

58. C. M. Belcher, J. M. Yearsley, R. M. Hadden, J. C. McElwain, G. Rein, Baseline intrinsic flammability of Earth's ecosystems estimated from paleoatmospheric oxygen over the past 350 million years. *Proc. Natl. Acad. Sci. U.S.A.* **107**, 22448–22453 (2010).

59. D. J. Over, Conodont biostratigraphy of the Chattanooga Shale, Middle and Upper Devonian, southern Appalachian Basin, eastern United States. *J. Paleo.* **81**, 1194–1217 (2007).

60. D. J. Over, E. Hauf, J. Wallace, J. Chiarello, J. S. Over, G. J. Gilleaudeau, Y. Song, T. J. Algeo, Conodont biostratigraphy and magnetic susceptibility of Upper Devonian Chattanooga Shale, eastern United States: Evidence for episodic deposition and disconformities. *Palaeogeogr. Palaeoclimatol. Palaeoecol.* **524**, 137–149 (2019).

61. J. Liu, T. J. Algeo, L. A. Hinnov, Identifying and quantifying stratigraphic disconformities in shale: An example from the Upper Devonian of the Appalachian Basin. *Geol. Soc. Am. Bull.* **137**, 1–15 (2025).

62. S. J. Baker, S. P. Hesselbo, T. M. Lenton, L. V. Duarte, C. M. Belcher, Charcoal evidence that rising atmospheric oxygen terminated Early Jurassic ocean anoxia. *Nat. Commun.* **8**, 15018 (2017).

63. A. C. Scott, The Pre-Quaternary history of fire. *Palaeogeogr. Palaeoclimatol. Palaeoecol.* **164**, 281–329 (2000).

64. M. J. Cope, W. G. Chaloner, Fossil charcoal as evidence of past atmospheric composition. *Nature* **283**, 647–649 (1980).

65. N. P. Rowe, T. P. Jones, Devonian charcoal. *Palaeogeogr. Palaeoclimatol. Palaeoecol.* **164**, 331–338 (2000).

66. V. A. Kravchinsky, Paleozoic large igneous provinces of Northern Eurasia: Correlation with mass extinction events. *Glob. Planet. Change* **86**, 31–36 (2012).

67. K. Norinaga, O. Deutschmann, N. Saegusa, J. I. Hayashi, Analysis of pyrolysis products from light hydrocarbons and kinetic modeling for growth of polycyclic aromatic hydrocarbons with detailed chemistry. *J. Anal. Appl. Pyrolysis* **86**, 148–160 (2009).

68. K. Kaiho, D. Tanaka, S. Richoz, D. S. Jones, R. Saito, D. Kameyama, M. Ikeda, S. Takahashi, M. Aftabuzzaman, M. Fujibayashi, Volcanic temperature changes modulated volatile release and climate fluctuations at the end-Triassic mass extinction. *Earth Planet. Sci. Lett.* **579**, 117364 (2022).

69. K. Kaiho, M. Miura, M. Tezuka, N. Hayashi, D. S. Jones, K. Oikawa, J. G. Casier, M. Fujibayashi, Z. Q. Chen, Coronene, mercury, and biomarker data support a link between extinction magnitude and volcanic intensity in the Late Devonian. *Glob. Planet. Change* **199**, 103452 (2021).

70. G. Racki, M. Rakociński, L. Marynowski, P. B. Wignall, Mercury enrichments and the Frasnian–Famennian biotic crisis: A volcanic trigger proved? *Geology* **46**, 543–546 (2018).

71. G. Racki, A volcanic scenario for the Frasnian–Famennian major biotic crisis and other Late Devonian global changes: More answers than questions? *Glob. Planet. Change* **189**, 103174 (2020).

72. J. Shen, T. J. Algeo, J. Chen, N. J. Planavsky, Q. Feng, J. Yu, J. Liu, Mercury in marine Ordovician/Silurian boundary sections of South China is sulfide-hosted and non-volcanic in origin. *Earth Planet. Sci. Lett.* **511**, 130–140 (2019).

73. J. Shen, Q. Feng, T. J. Algeo, J. Liu, C. Zhou, W. Wei, J. Liu, T. R. Them II, B. C. Gill, J. Chen, Sedimentary host phases of mercury (Hg) and implications for use of Hg as a volcanic proxy. *Earth Planet. Sci. Lett.* **543**, 116333 (2020).

74. I. J. Glasspool, A. C. Scott, D. Waltham, N. Pronina, L. Shao, The impact of fire on the Late Paleozoic Earth system. *Front. Plant Sci.* **6**, 756 (2015).

75. M. M. Joachimski, W. Buggisch, Conodont apatite $\delta^{18}O$ signatures indicate climatic cooling as a trigger of the Late Devonian mass extinction. *Geology* **30**, 711–714 (2002).

76. T. J. Algeo, M. N. Remírez, H. Zhao, L. Schwark, G. Gordon, A. Anbar, S. Bates, T. Lyons, D. J. Over, B. Sageman, Transient glacio-eustatic fall and its climato-environmental effects during the Frasnian-Famennian transition. *Glob. Planet. Change* **256**, 105135 (2026).

77. M. Lu, Y. Lu, T. Ikejiri, D. Sun, R. Carroll, E. H. Blair, T. J. Algeo, Y. Sun, Periodic oceanic euxinia and terrestrial fluxes linked to astronomical forcing during the Late Devonian Frasnian–Famennian mass extinction. *Earth Planet. Sci. Lett.* **562**, 116839 (2021).

78. G. Le Hir, Y. Donnadieu, Y. Goddéris, B. Meyer-Berthaud, G. Ramstein, R. C. Blakey, The climate change caused by the land plant invasion in the Devonian. *Earth Planet. Sci. Lett.* **310**, 203–212 (2011).

79. S. Carr, A. Boom, H. L. Grimes, B. M. Chase, M. E. Meadows, A. Harris, Leaf wax *n*-alkane distributions in arid zone South African flora: Environmental controls, chemotaxonomy and palaeoecological implications. *Org. Geochem.* **67**, 72–84 (2014).

80. D. Sachse, J. Radke, G. Gleixner, δD values of individual *n*-alkanes from terrestrial plants along a climatic gradient–Implications for the sedimentary biomarker record. *Org. Geochem.* **37**, 469–483 (2006).

81. R. T. Bush, F. A. McInerney, Leaf wax *n*-alkane distributions in and across modern plants: Implications for paleoecology and chemotaxonomy. *Geochim. Cosmochim. Acta* **117**, 161–179 (2013).

82. B. Hoffmann, A. Kahmen, L. A. Cernusak, S. K. Arndt, D. Sachse, Abundance and distribution of leaf wax *n*-alkanes in leaves of Acacia and Eucalyptus trees along a strong humidity gradient in northern Australia. *Org. Geochem.* **62**, 62–67 (2013).

83. J. Liu, J. Zhao, D. He, X. Huang, C. Jiang, H. Yan, G. Lin, Z. An, Effects of plant types on terrestrial leaf wax long-chain *n*-alkane biomarkers: Implications and paleoapplications. *Earth Sci. Rev.* **235**, 104248 (2022).

84. B. Cascales-Miñana, Apparent changes in the Ordovician–Mississippian plant diversity. *Rev. Palaeobot. Palynol.* **227**, 19–27 (2016).

85. P. Giesen, C. M. Berry, Reconstruction and growth of the early tree *Calamophyton* (Pseudosporochnales, Cladoxylopsida) based on exceptionally complete specimens from Lindlar, Germany (Mid-Devonian): Organic connection of *Calamophyton* branches and *Duisbergia* trunks. *Int. J. Plant Sci.* **174**, 665–686 (2013).

86. C. M. Berry, J. E. Marshall, Lycopsid forests in the early Late Devonian paleoequatorial zone of Svalbard. *Geology* **43**, 1043–1046 (2015).

87. W. E. Stein, C. M. Berry, J. L. Morris, L. V. Hernick, F. Mannolini, C. Ver Straeten, E. Landing, J. E. Marshall, C. H. Wellman, D. J. Beerling, Mid-Devonian *Archaeopteris* roots signal revolutionary change in earliest fossil forests. *Curr. Biol.* **30**, 421–431.e2 (2020).

88. N. S. Davies, W. J. McMahon, C. M. Berry, Earth's earliest forest: Fossilized trees and vegetation-induced sedimentary structures from the Middle Devonian (Eifelian) Hangman Sandstone Formation, Somerset and Devon, SW England. *J. Geol. Soc. London* **181**, jgs2023–2204 (2024).

89. R. P. Curry, Miospores from the Upper Devonian (Frasnian) Greenland Gap Group, Allegheny Front, Maryland, West Virginia and Virginia, USA. *Rev. Palaeobot. Palynol.* **20**, 119–131 (1975).

90. J. E. Marshall, P. F. Holterhoff, S. R. El-Abdallah, K. K. Matsunaga, A. W. Bronson, A. M. Tomescu, The Archaeopterid forests of Lower Frasnian (Upper Devonian) westernmost Laurentia: Biota and depositional environment of the Maywood Formation in northern Wyoming as reflected by palynoflora, macroflora, fauna, and sedimentology. *Int. J. Plant Sci.* **183**, 465–492 (2022).

91. J. E. Marshall, T. R. Astin, O. P. Tel'nova, P. Gaca, Terrestrial palaeoclimate, mercury, atmospheric CO_2 and land plants through the Late Devonian mass extinction. *J. Geol. Soc. London* **182**, jgs2024–2187 (2025).

92. L. C. Conant, V. E. Swanson, Chattanooga Shale and related rocks of central Tennessee and nearby areas. *U.S. Geol. Surv. Prof. Pap.* **357**, 1–91 (1961).

93. S. R. de la Rue, H. D. Rowe, S. M. Rimmer, Palynological and bulk geochemical constraints on the paleoceanographic conditions across the Frasnian–Famennian boundary, New Albany Shale, Indiana. *Int. J. Coal Geol.* **71**, 72–84 (2007).

94. Y. Li, J. Schieber, On the origin of a phosphate enriched interval in the Chattanooga Shale (Upper Devonian) of Tennessee—A combined sedimentologic, petrographic, and geochemical study. *Sediment. Geol.* **329**, 40–61 (2015).

95. P. Aharon, Redox stratification and anoxia of the early Precambrian oceans: Implications for carbon isotope excursions and oxidation events. *Precambrian Res.* **137**, 207–222 (2005).

96. B. Xu, Z. Gu, C. Wang, Q. Hao, J. Han, Q. Liu, L. Wang, Y. Lu, Carbon isotopic evidence for the associations of decreasing atmospheric CO_2 level with the Frasnian–Famennian mass extinction. *J. Geophys. Res.-Biogeosci.* **117**, G01032 (2012).

97. J. D. Owens, T. W. Lyons, C. M. Lowery, Quantifying the missing sink for global organic carbon burial during a Cretaceous oceanic anoxic event. *Earth Planet. Sci. Lett.* **499**, 83–94 (2018).

98. D.C. Catling, "6.7-The great oxidation event transition" in *Treatise on Geochemistry*, H. D. Holland, K. K. Turekian, Eds. (Elsevier, 2014), vol. 6, pp. 177–195.

99. J. Golonka, Late Devonian paleogeography in the framework of global plate tectonics. *Glob. Planet. Change* **186**, 103129 (2020).

100. R. T. Becker, P. Königshof, C. E. Brett, Devonian climate, sea level and evolutionary events: An introduction. *Geol. Soc. Lond. Spec. Publ.* **423**, 1–10 (2016).

101. Z. Liu, D. Selby, P. C. Hackley, D. J. Over, Evidence of wildfires and elevated atmospheric oxygen at the Frasnian–Famennian boundary in New York (USA): Implications for the Late Devonian mass extinction. *Geol. Soc. Am. Bull.* **132**, 2043–2054 (2020).

102. C. Huang, M. M. Joachimski, Y. Gong, Did climate changes trigger the Late Devonian Kellwasser Crisis? Evidence from a high-resolution conodont $\delta^{18}O_{PO4}$ record from South China. *Earth Planet. Sci. Lett.* **495**, 174–184 (2018).

103. R. A. Berner, Z. Kothavala, GEOCARB III: A revised model of atmospheric CO_2 over Phanerozoic time. *Am. J. Sci.* **301**, 182–204 (2001).

104. M. Joachimski, S. Breisig, W. Buggisch, J. Talent, R. Mawson, M. Gereke, J. Morrow, J. Day, K. Weddige, Devonian climate and reef evolution: Insights from oxygen isotopes in apatite. *Earth Planet. Sci. Lett.* **284**, 599–609 (2009).

105. M. T. Hurtgen, S. B. Pruss, A. H. Knoll, Evaluating the relationship between the carbon and sulfur cycles in the later Cambrian ocean: An example from the Port au Port Group, western Newfoundland, Canada. *Earth Planet. Sci. Lett.* **281**, 288–297 (2009).

106. R. Becker, J. Marshall, A.C. Da Silva, F. Agterberg, F. Gradstein, J. Ogg, "Chapter 22 - The Devonian Period," in *Geologic time scale 2020*, F. M. Gradstein, J. G. Ogg, M. D. Schmitz, G. M. Ogg, Eds. (Elsevier, 2020), pp. 733–810.

107. S. K. Carmichael, J. A. Waters, P. Königshof, T. J. Suttner, E. Kido, Paleogeography and paleoenvironments of the Late Devonian Kellwasser event: A review of its sedimentological and geochemical expression. *Glob. Planet. Change* **183**, 102984 (2019).

108. G. Klapper, R. Feist, R. T. Becker, M. R. House, Definition of the Frasnian/Famennian stage boundary. *Episodes* **16**, 433–441 (1993).

109. D. J. Over, The Frasnian/Famennian boundary in central and eastern United States. *Palaeogeogr. Palaeoclimatol. Palaeoecol.* **181**, 153–169 (2002).

110. M. T. Whalen, M. G. Śliwiński, J. H. Payne, J. E. Day, D. Chen, A. C. Da Silva, Chemostratigraphy and magnetic susceptibility of the Late Devonian Frasnian–Famennian transition in western Canada and southern China: Implications for carbon and nutrient cycling and mass extinction. *Geol. Soc. Lond. Spec. Publ.* **414**, 37–72 (2015).

111. J. J. Brocks, G. D. Love, R. E. Summons, A. H. Knoll, G. A. Logan, S. A. Bowden, Biomarker evidence for green and purple sulphur bacteria in a stratified Palaeoproterozoic sea. *Nature* **437**, 866–870 (2005).

112. J. J. Brocks, P. Schaeffer, Okenane, a biomarker for purple sulfur bacteria (*Chromatiaceae*), and other new carotenoid derivatives from the 1640 Ma Barney Creek Formation. *Geochim. Cosmochim. Acta* **72**, 1396–1414 (2008).

113. K. French, D. Rocher, J. Zumberge, R. Summons, Assessing the distribution of sedimentary C_{40} carotenoids through time. *Geobiology* **13**, 139–151 (2015).
